# Inter-Limb Muscle Asymmetries in Youth Athletes: A Comprehensive Systematic Review and Meta-Analysis of Single-Leg Jump and Change of Direction Speed Outcomes

**DOI:** 10.3390/sports14070283

**Published:** 2026-07-06

**Authors:** Adam Maszczyk, Mariola Gepfert, Przemysław Pietraszewski, Anna Zwierzchowska, Adam Zając

**Affiliations:** Institute of Sport Sciences, Academy of Physical Education, 41-065 Katowice, Poland; m.gepfert@awf.katowice.pl (M.G.); a.zajac@awf.katowice.pl (A.Z.)

**Keywords:** inter-limb asymmetry, youth athletes, maturation

## Abstract

The aim of this meta-analysis was to synthesize current evidence on inter-limb asymmetries in youth athletes and to determine their magnitude, developmental determinants, and functional relevance. The review followed PRISMA 2020 guidelines, and the protocol was registered in PROSPERO. Six databases were searched from inception to October 2025. Studies assessing asymmetry as a between-limb difference in athletes aged 6–18 years were included. A total of 25 studies (*N* = 4125) were included qualitatively, with 24 included in the quantitative analyses. Meta-analyses were conducted for comparable outcomes (single-leg countermovement jump [SLCMJ], change of direction speed [COD], association with sprint performance, and maturation effects) using random-effects models and heterogeneity assessment (I^2^, τ^2^). Mean asymmetry was 10.8% for SLCMJ (95% CI: 6.7–14.9; I^2^ = 78%) and 7.4% for COD (95% CI: 0.5–14.2; I^2^ = 64%). The association between asymmetry and sprint performance was small and not statistically significant (r = −0.27; 95% CI: −0.55 to 0.07). Maturation analysis showed a moderate effect (d = 0.35; 95% CI: 0.18–0.52), with peak asymmetry around peak height velocity (PHV). Heterogeneity was mainly explained by sport-specific demands and methodological differences. Asymmetries of approximately 10% are commonly observed in youth athletes in single-leg jump and change of direction tests, but their clinical relevance likely depends on sport-specific demands, maturation status, and testing modality, and should not be interpreted as a universal normative threshold. The lack of prospective injury data prevents the establishment of universal clinical thresholds. In conclusion, inter-limb asymmetries are common and developmentally dynamic in youth athletes, with functional relevance depending on biological and sport-specific context. Future research should prioritize methodological standardization and prospective designs.

## 1. Introduction

Inter-limb muscle asymmetry, broadly understood as systematic between-limb differences in strength, power, movement mechanics, morphology, or neuromuscular control, is frequently reported in young athletes and is often considered an expected consequence of sport participation. Unilateral or rotational actions, such as kicking, cutting, jumping off a preferred leg, throwing, or striking, can progressively bias loading patterns toward one side, while bilateral actions can still be executed with functional dominance [1,2,3]. In this context, small asymmetries can represent normal, sport-specific adaptation. However, when asymmetries become large, persistent, or coupled with deficient movement control, they may reflect maladaptive neuromuscular profiles that are plausibly linked to reduced performance efficiency and elevated injury susceptibility, particularly in high-load tasks involving rapid deceleration, landing, or change of direction [3,4]. Distinguishing between physiological, functional, and potentially problematic asymmetry is therefore not straightforward and cannot be resolved by applying a single magnitude threshold. Whether a given between-limb difference represents a normal sport-specific adaptation, a functionally compensated difference that does not impair performance, or a genuinely maladaptive asymmetry warranting intervention depends on at least five contextual factors: (i) the specific test used and the neuromuscular quality it captures; (ii) the technical demands of the athlete’s sport; (iii) the athlete’s biological maturation status relative to PHV; (iv) the individual’s training and injury history; and (v) whether the asymmetry is associated with measurable deficits in functional performance outcomes [3]. This multi-factorial interpretive framework is central to the present review and motivates the structured approach to evidence synthesis adopted here.

In youth populations, the interpretation of asymmetry is uniquely challenging because asymmetry is superimposed on rapid biological growth and maturation. During the transition into and through puberty, increases in limb length, body mass, and segment inertia, together with transient disruptions in coordination, can modify bilateral force sharing and inter-limb control in a manner that is not fully captured by chronological age alone [5]. Peak height velocity (PHV) is therefore increasingly used to contextualize neuromuscular development because it reflects individual differences in biological timing and tempo. From an applied perspective, this maturation-related variability complicates the use of a single “acceptable” asymmetry value across broad age groups, and it may also explain why the same asymmetry magnitude could be benign in one developmental stage but concerning in another.

A further obstacle to evidence-based interpretation is the pronounced heterogeneity in how asymmetry is defined and measured. Studies quantify asymmetry using a range of indices (percentage difference, limb symmetry index, symmetry angle, absolute difference) across distinct tests that target different neuromuscular qualities (e.g., single-leg countermovement jump, hop tests, isokinetic dynamometry, balance tasks, or sport-specific change of direction protocols). Even within the same test family, methodological differences (force plate vs. field-based measures, jump height vs. impulse vs. peak force, deficit-based vs. time-based change of direction metrics) may yield different asymmetry estimates and different relationships with performance. Consequently, the literature contains a large number of sport- and test-specific observations but comparatively few quantitative syntheses capable of separating consistent patterns from context-dependent variability.

Existing research suggests that sport discipline is a major determinant of asymmetry phenotype. Sports with inherently unilateral technical demands (e.g., tennis or striking/martial disciplines) tend to display more pronounced side-specific structural and functional adaptations, whereas many team sports exhibit smaller mean asymmetries but wide inter-individual variability driven by positional roles, preferred limb usage, and accumulated exposure to unilateral skills [6,7,8,9]. At the same time, the practical question faced by coaches and clinicians is not simply whether asymmetry exists, but how large asymmetry typically is in specific youth sport contexts, how it evolves across the maturational continuum, and how it relates to the performance demands of the sport (e.g., sprint acceleration, repeated change of direction ability, or explosive unilateral propulsion). Although thresholds such as 10% are commonly cited in applied settings [3], the empirical basis for universal cutoffs in youth remains uncertain, especially given the scarcity of prospective injury studies, the predominance of cross-sectional designs, and the variability in testing approaches.

Against this background, a systematic review with meta-analysis can add value in two complementary ways. First, it can provide pooled estimates of asymmetry magnitude for the most commonly reported and methodologically comparable tests, thereby offering evidence-informed reference ranges and quantifying between-study heterogeneity. Second, where sufficient data exist, it can quantify the direction and magnitude of associations between asymmetry and functional performance outcomes, and explore whether sport demands or maturational stage plausibly modify these relationships. Importantly, however, the ability to derive “clinical thresholds” for injury prediction depends on the availability of robust prospective injury surveillance with consistent exposure and outcome definitions. Because such data are limited in youth asymmetry research and the included evidence is predominantly observational and cross-sectional, the most defensible objective of meta-analysis in this field is to synthesize reference magnitudes and performance associations, not to assert universal injury risk cutoffs. Therefore, any practical interpretation must remain explicitly conditional on sport, test type, and maturation context.

Accordingly, the purpose of the present work was to systematically synthesize the evidence on inter-limb muscle asymmetries in youth athletic populations and to quantify, using meta-analytic methods where feasible, (i) the magnitude of lower limb asymmetry in commonly used unilateral jump assessments, (ii) the magnitude of asymmetry in change of direction tasks, (iii) the association between asymmetry and linear sprint performance, and (iv) the extent to which maturation status (especially PHV classification when available) modifies asymmetry expression. The secondary aim was to evaluate whether sport-demand classification (predominantly unilateral/rotational vs. predominantly bilateral/team sport demands) and participant characteristics (sex and competitive level) could help explain heterogeneity in asymmetry estimates. Finally, by integrating quantitative pooling with structured narrative synthesis for outcomes not suitable for meta-analysis, this review aims to clarify what is currently supported by evidence, where conclusions remain uncertain due to methodological heterogeneity or limited study designs, and which research gaps, particularly prospective injury studies with standardized asymmetry metrics, must be addressed before reliable, context-specific clinical decision thresholds can be established in youth sport practice.

## 2. Materials and Methods

### 2.1. Study Design and Protocol Registration

This work was designed as a systematic review with quantitative synthesis conducted wherever the available evidence was sufficiently comparable to justify pooling. The review protocol was developed a priori in accordance with PRISMA 2020 recommendations and registered prospectively in PROSPERO (CRD420261294739). The analytic workflow (effect size calculation, domain grouping, and primary models) followed the preregistered PROSPERO protocol, with deviations (non-use of meta-regression due to insufficient study counts and non-reporting of prediction intervals) explicitly documented in the Results section. Any deviations from the protocol (primarily restrictions introduced post hoc to prevent inappropriate pooling of heterogeneous outcomes) are explicitly described in the Section 2.11 below, together with the rationale for those decisions. Two reviewers independently conducted all stages of screening, extraction, and quality appraisal, with disagreements resolved by consensus and, when necessary, by adjudication by a third reviewer. The completed PRISMA 2020 checklist is provided in the Supplementary Material (Table S1).

### 2.2. Information Sources and Search Strategy

A comprehensive literature search was performed in MEDLINE (via PubMed), Scopus, Web of Science Core Collection, Semantic Scholar, SPORTDiscus, and OpenAlex from database inception to October 2025. To reduce the risk of missing relevant datasets, reference lists of included studies and relevant reviews were screened manually, and preprint servers and gray literature sources were also checked to identify potentially relevant records. The search combined controlled vocabulary (e.g., MeSH terms where available) and free-text keywords related to asymmetry and laterality (e.g., “inter limb asymmetry”, “bilateral imbalance”, “limb symmetry index”), youth populations (“child”, “adolescent”, “youth”, “junior athlete”), and sport/performance outcomes (“sport”, “athlete”, “sprint”, “jump”, “change of direction”, “injury”). The complete search syntax for each database, together with date limits and applied filters, is provided in the Supplementary Material (Table S5).

### 2.3. Eligibility Criteria

Studies were eligible if they included athletes aged 6–18 years who participated in organized sport and reported a quantitative measure of inter-limb asymmetry in a neuromuscular, biomechanical, morphological, or postural variable relevant to athletic performance. Eligible study designs included peer-reviewed observational studies (cross-sectional, cohort, or longitudinal) and randomized controlled trials when extractable pre-intervention baseline asymmetry data were available. Only full-text articles published in English were included.

Studies were excluded if they focused exclusively on adults or clinical pediatric populations without a separable healthy youth athlete subgroup, did not report extractable asymmetry data or measures of dispersion, were case reports, case series with fewer than ten participants, conference abstracts, editorials, narrative reviews, or non-peer-reviewed gray literature, or involved para-athlete cohorts in whom asymmetry primarily reflected impairment rather than sport-related adaptation. When multiple reports described the same cohort, the most comprehensive report was retained. These criteria were predefined according to the PICOS framework.

### 2.4. PICOS Framework

The review question was structured according to PICOS: population, youth athletes aged 6–18 years; exposure, inter-limb asymmetry assessment; comparison, between-limb and, where available, between-group contrasts; outcomes, asymmetry magnitude and performance-related associations; and study design, peer-reviewed observational studies and randomized trials with extractable baseline data.

### 2.5. Outcomes and Operational Definition of Asymmetry

Studies were eligible for the systematic review if they quantified inter-limb asymmetry as a between-limb difference in a measurable neuromuscular, biomechanical, or anthropometric variable. Because asymmetry can be operationalized in multiple ways, a harmonized metric was applied for quantitative synthesis. When possible, asymmetry magnitude was recalculated as an absolute percentage difference using the formula asymmetry (%) = 100 × |L − R|/max (L, R), where L and R represent left and right limb values [3]. When the limb symmetry index (LSI) was reported as (weaker/stronger) × 100, it was converted to asymmetry as 100 − LSI [3]. If studies reported directional asymmetry (dominant vs non-dominant), absolute magnitude values were used for pooling because dominance definitions varied across sports and were frequently based on self-report rather than standardized criteria.

To directly address methodological heterogeneity, meta-analyses were restricted to outcome domains with sufficiently comparable constructs and metrics. Quantitative pooling was conducted only for lower limb asymmetry expressed as percentages (or convertible to percentages) derived from unilateral vertical jump tests classified as single-leg countermovement jump (SLCMJ) or equivalent unilateral CMJ outputs, for time-based change of direction (COD) asymmetry quantified using total completion time, and for associations between asymmetry and linear sprint performance reported as correlation coefficients. Imaging-derived morphological asymmetry (DXA/MRI), isolated isokinetic strength asymmetry, stabilometric postural measures, and iso-inertial variables were included in the systematic review but were synthesized narratively because methodological diversity and sparse study counts prevented defensible quantitative pooling. Deficit-based COD measures (e.g., COD deficit calculated by subtracting linear sprint time from COD time) were treated as conceptually distinct from total time asymmetry and were therefore not pooled together with time-based COD asymmetry estimates.

In this review, the term inter-limb asymmetry is used to denote quantitative between-limb differences in measurable neuromuscular, biomechanical, morphological, or postural variables. The term imbalance is used only descriptively to refer to the same between-limb differences and does not imply a distinct construct or diagnosis. Laterality refers to side preference (e.g., preferred kicking leg or racket arm) and is treated as an explanatory characteristic that may underpin observed asymmetries rather than as an outcome variable. Dominance is used in the conventional sport science sense to describe the limb designated as dominant (preferred leg/arm), but all meta-analytic models were based on the absolute magnitude of between-limb differences, irrespective of the dominant side.

### 2.6. Study Selection Process

After exporting the search results to reference management software, duplicates were removed prior to screening. Titles and abstracts were screened independently by two reviewers, and records were subjected to full-text review when either reviewer judged them to be potentially eligible. Full texts were then assessed independently using a standardized eligibility form. Reasons for exclusion at the full-text stage were recorded and are reported in the PRISMA flow diagram, with a complete list of full-text records excluded together with the primary reason for exclusion provided as Supplementary Material (Table S2). This two-stage, dual-reviewer process was used to minimize selection bias and to ensure that inclusion decisions were reproducible. Potential duplicate or overlapping cohorts were assessed by cross-checking study identifiers (authors, institutions, and sample sizes), recruitment periods, and participant characteristics across all included records, with particular attention to soccer and handball studies from the same research groups. When multiple reports described the same or an overlapping cohort, we treated them as a single dataset for meta-analytic purposes and retained the most complete or methodologically informative record, using supplementary reports only for additional descriptive information. These decisions are documented in the study selection log and are reflected in the per-study characteristics table.

### 2.7. Sport Demand Classification

Prior to data extraction, all sports represented in the included studies were classified into two pre-specified demand categories: (i) predominantly unilateral/rotational sports, defined as disciplines in which the primary technical actions (e.g., kicking, striking, throwing, or racket play) are performed consistently with one preferred limb, creating systematic side-specific loading, and (ii) predominantly bilateral/team-based sports, defined as disciplines in which competitive actions involve both limbs in alternating or symmetric roles, even if individual players develop positional preferences. This classification was defined a priori in the PROSPERO protocol. The assignments were as follows: tennis, taekwondo, and cricket were classified as unilateral/rotational; soccer, handball, volleyball, basketball, football, badminton, ice hockey, and athletics were classified as bilateral/team-based. Studies that recruited athletes from multiple sports simultaneously (mixed sport cohorts) were not assigned to either category and were excluded from sport-demand subgroup comparisons; their data contributed only to the overall pooled estimates and narrative synthesis. Any ambiguous classification was resolved by consensus between the two reviewers prior to data extraction, with disagreements adjudicated by a third reviewer.

### 2.8. Data Extraction

Data extraction was performed independently by two reviewers using a piloted extraction template. Extracted variables included study identifiers (authors, year, country), study design, sport discipline, competition level, sample size, sex distribution, age and maturation status (including PHV classification when reported), testing protocols, asymmetry computation method, and reliability information (ICC, CV) when available. Outcome data extracted for quantitative synthesis included mean asymmetry values with measures of dispersion (SD, SE, or CI), sprint and COD performance values, and correlation coefficients between asymmetry and performance outcomes. When dispersion data were not directly reported, they were derived from available statistics (confidence intervals, standard errors, t-values, or *p*-values) using standard conversions; if derivation was not possible, the study remained eligible for narrative synthesis but was excluded from the relevant meta-analysis. To avoid unit of analysis errors, only one effect estimate per study and per meta-analytic domain was included in the primary model. When a study reported multiple sprint distances, a pre-specified hierarchy was used to enhance comparability across studies, prioritizing the most commonly available distance (20 m, followed by 10 m and 30 m). When studies reported separate, non-overlapping subgroups (independent male and female cohorts), subgroup estimates were either combined into a single study-level estimate using standard pooling equations or included separately only when independence was ensured.

For single-leg countermovement jump (SLCMJ) and change of direction (COD) assessments, multiple outcome metrics were frequently reported within the same study. To ensure consistency and comparability across studies, we applied a predefined hierarchy of preferred and alternative outcomes.

For SLCMJ, the primary outcome was the percentage inter-limb asymmetry in jump height (or flight time-derived height), expressed as the absolute percentage difference between limbs. When jump height was not available, we used alternative metrics in the following order of preference: peak propulsive force, peak power, or countermovement depth-normalized force, provided that inter-limb asymmetry could be derived as a percentage index.

For COD tests, the primary outcome was the percentage inter-limb asymmetry in time-based performance (e.g., completion time of left vs right leg plant trials) for the same COD test configuration. When more than one COD test was available, we prioritized tasks most commonly used in the literature (e.g., 505 test, modified 505 test, pro-agility shuttle), selecting the configuration reported for both limbs under comparable conditions. If only directional asymmetry (dominant vs non-dominant) was reported, we used the absolute magnitude of the reported asymmetry index.

When multiple metrics within the same construct were reported (both raw between-limb differences and limb symmetry index), we extracted the metric that could be most transparently converted into a common, absolute percentage asymmetry. All decision rules were applied consistently across studies and documented during data extraction.

### 2.9. Risk of Bias Assessment

Risk of bias was assessed using design-appropriate tools rather than a single scale across heterogeneous study types. Analytical cross-sectional studies were appraised using the Joanna Briggs Institute (JBI) critical appraisal checklist for cross-sectional studies, cohort studies using the JBI cohort checklist, and randomized trials (when baseline data were extracted) using RoB 2. Each study was judged across domains relevant to asymmetry research, including participant selection, measurement validity and reliability, handling of confounding factors (particularly age, sex, maturation, and training exposure), and completeness of outcome reporting. Domain-level judgments were summarized at the study level, and disagreements were resolved by consensus. Per study, risk-of-bias judgments for each included study are provided in the Supplementary Material (Table S3). Domain-level risk of bias assessments for all included studies are summarized in Supplementary Table S3, which presents item-specific judgments for each JBI or RoB 2 domain. These procedures replaced earlier reliance on a single Newcastle–Ottawa Scale approach for all designs, because design-specific instruments provide clearer domain mapping and better interpretability across cross-sectional and cohort structures.

### 2.10. Certainty of Evidence

To provide a formal appraisal of confidence in pooled estimates, certainty of evidence was evaluated at the outcome level using the GRADE framework. Because the evidence base is predominantly observational, certainty ratings began at “low” and could be downgraded for risk of bias, inconsistency (including unexplained heterogeneity), indirectness (mismatch between test construct and sport demands), imprecision (wide confidence intervals and limited information size), and suspected publication bias. Upgrading was considered only when effects were large and consistent with plausible dose-response patterns. GRADE evidence profiles were prepared for each quantitative domain to ensure that conclusions and practical implications remained aligned with the strength of evidence, and the resulting Summary-of-Findings table is provided in the Supplementary Material (Table S4). Supplementary Table S4 reports domain-specific GRADE judgments (risk of bias, inconsistency, indirectness, imprecision, publication bias) and overall certainty ratings for each pooled outcome.

### 2.11. Data Synthesis and Statistical Analysis

All included studies contributed to the qualitative synthesis, which used structured tables to summarize sport-specific asymmetry magnitudes, developmental trends, and associations with performance and injury outcomes. For quantitative synthesis, studies were prospectively grouped by outcome construct. Pooling was restricted to four pre-specified, methodologically comparable domains: (a) single-leg countermovement jump (SLCMJ) asymmetry expressed as a percentage; (b) time-based change of direction (COD) asymmetry expressed as a percentage; (c) associations between SLCMJ asymmetry and linear sprint performance, expressed as Pearson correlation coefficients; and (d) standardized contrasts in asymmetry magnitude across biological maturation stages (pre-PHV, circa-PHV, post-PHV), or comparable chronological age strata. Outcomes that were conceptually distinct or methodologically heterogeneous, including imaging-derived morphological asymmetry (DXA/MRI/ultrasound), isolated isokinetic strength asymmetry, stabilometric postural asymmetry, iso-inertial crossover metrics, and deficit-based COD asymmetry, were synthesized narratively and not combined with the time-based COD pool.

The decision to include an outcome domain in the quantitative pooling was based on three pre-specified criteria, all of which had to be met simultaneously: (i) the construct measured had to be conceptually equivalent across studies (e.g., all studies measuring the same neuromuscular quality using the same test family); (ii) the effect size metric had to be expressible in a common unit without requiring assumptions that could distort the estimate (percentage asymmetry, Pearson’s r, or Hedges’ g); and (iii) a minimum of three studies with sufficient data for meta-analytic computation had to be available. Outcomes that failed to meet any one of these criteria were retained in the systematic review but assigned to narrative synthesis. Imaging-derived morphological asymmetry (DXA/MRI/ultrasound), isolated isokinetic strength asymmetry, stabilometric postural asymmetry, iso-inertial crossover metrics, and deficit-based COD asymmetry each failed at least one criterion, most commonly conceptual nonequivalence across instruments or an insufficient number of methodologically comparable studies, and were therefore not pooled.

To avoid dependence among effect sizes, only one effect estimate per study was included in each meta-analytic model. When a study reported multiple eligible outcomes within the same domain (several sprint distances, multiple SLCMJ variables, or sex-specific subgroups), predefined selection rules were applied; a single primary metric was selected according to the pre-specified hierarchy described in the Data Extraction section, or when a study reported truly independent, non-overlapping subgroups (separate male and female cohorts with no participants in common), subgroup estimates were combined into a single study-level effect using standard inverse variance pooling formulas before entry into the model. Age group or maturation-stage contrasts within a single study were treated as a single study-level standardized mean difference (Hedges’ g) computed from the most extreme available contrast (pre-PHV vs. post-PHV) or pooled across adjacent contrasts when more than two stages were reported, and raw data or sufficient summary statistics were available. Multilevel models and robust variance estimation were not used because this one-effect-per study restriction was enforced a priori, rendering within-study dependence structurally impossible in primary models.

For pooling of asymmetry magnitudes expressed as percentages, meta-analysis of means was performed using inverse variance weighting. A meta-analysis of means (rather than of standardized mean differences) was selected because the target estimand was the absolute magnitude of inter-limb asymmetry expressed on a common, clinically interpretable percentage scale, which is the metric used in applied practice and in the threshold literature; standardizing these values would have obscured the very quantity of interest. Because percentage asymmetry is a bounded ratio that can be right-skewed, two considerations were addressed. First, the inverse variance estimator does not assume normally distributed raw observations; it requires only approximately normal sampling distributions of the study-level means, which is supported by the moderate to large samples in most of the included studies (Central Limit Theorem). Second, the appropriateness of variance-stabilizing transformations (logarithmic or arcsine) was considered but not adopted because back transformation would have reintroduced interpretation on a non-percentage scale and because the observed study-level means were not concentrated near the natural boundary at zero. For the correlation domain, Fisher’s z transformation was applied precisely as a variance-stabilizing step, with pooling in z-space and back transformation for reporting. As a check on distributional assumptions, the leave-one-out and influence diagnostics described below were used to confirm that no single (potentially skewing) study governed pooled estimates. For sprint performance associations, Pearson’s correlation coefficients were transformed using Fisher’s z to stabilize variance, pooled in z-space, and back-transformed for interpretation. For maturation-related comparisons, standardized mean differences (Hedges’ g) were calculated when studies provided sufficient data for contrasts between maturation stages (pre-PHV vs. circa-PHV, circa-PHV vs post-PHV). When repeated measures designs were present but within-person correlations were not reported, effect sizes were calculated conservatively using available summary statistics and interpreted cautiously as complementary to the broader narrative synthesis.

Where reported by the original studies, maturity offset (years from PHV) was extracted and summarized in Table 1. When numerical maturity offset values were unavailable, the reported maturational category or status descriptor was retained; otherwise, it was indicated as not reported (NR).

Because clinical and methodological diversity was expected even within restricted domains (differences in sports, participant characteristics, instrumentation, and protocols), random-effects models were specified as the default approach for all meta-analyses rather than selecting fixed versus random effects solely based on I^2^ thresholds. Between-study variance (τ^2^) was estimated using restricted maximum likelihood (REML). To improve inference in meta-analyses with a small number of studies, Hartung–Knapp adjustments were applied to confidence intervals where applicable. Although 95% prediction intervals were considered at the planning stage, they were ultimately not reported because the number of studies per domain was generally small, and estimates were judged to be too imprecise for meaningful interpretation. Statistical heterogeneity was quantified using Cochran’s Q and I^2^, but interpretation focused on the substantive sources of variability rather than threshold-based model switching.

Planned subgroup analyses were intentionally restricted to avoid underpowered comparisons and were conducted only when there were at least four studies per subgroup and the underlying constructs and metrics were directly comparable. Otherwise, potential moderators such as sport-demand classification, sex, maturation stage, and testing approach were explored descriptively within the narrative synthesis. Meta-regression was pre-specified in the PROSPERO protocol as a non-primary option but, given the limited number of studies per potential moderator and the risk of unstable estimates, no formal meta-regression models were ultimately fitted; heterogeneity exploration therefore relied on structured qualitative comparisons rather than regression modeling. To explore the substantial heterogeneity observed in the percentage asymmetry domains, formal influence diagnostics were computed for each primary model using the metafor package: leave-one-out (LOO) estimation, in which the pooled effect and I^2^ were recomputed with each study omitted in turn; Baujat plots, which display each study’s contribution to overall heterogeneity against its influence on the pooled estimate; and standard case deletion influence statistics (including studentized residuals, DFFITS, Cook’s distance, and leave-one-out estimates of τ^2^). These diagnostics were used to determine whether the heterogeneity and the pooled estimates were driven by any single study and to confirm the robustness of the synthesis; their results are reported in the Meta-Analysis Results and summarized in the Supplementary Material. Sensitivity analyses included leave-one-out diagnostics, restriction to studies judged to have a lower risk of bias, and restriction to studies using directly reported (non-converted) asymmetry metrics to examine the impact of the harmonization procedures. Annotated statistical code used for all meta-analytic models will be made available in an open repository upon publication to facilitate transparency and reproducibility.

### 2.12. Assessment of Publication Bias and Small-Study Effects

Publication bias and small-study effects were assessed in line with contemporary recommendations, acknowledging that formal tests have low power when the number of studies is limited [33]. Funnel plots and Egger’s regression [34] were planned only for meta-analyses with at least ten studies. For domains with fewer studies, publication bias was addressed qualitatively within the GRADE framework by examining whether effect estimates differed systematically by sample size, checking for selective outcome reporting within studies (mismatch between reported methods and outcomes), and interpreting pooled effects cautiously when imprecision and heterogeneity were substantial.

### 2.13. Software and Reporting

All quantitative meta-analyses were conducted in R (version 4.3.3; R Foundation for Statistical Computing, Vienna, Austria) using the metafor package. Random-effects models with restricted maximum likelihood (REML) estimation were used to pool effect sizes for percentage inter-limb asymmetry and correlation coefficients. Where necessary, reported statistics (means, standard deviations, confidence intervals, *p*-values, or correlation coefficients) were converted into a common effect size metric following standard formulas appropriate for each outcome type. For key primary models, results were cross-checked in Stata (version 18.0; StataCorp LLC., College Station, TX, USA) to verify model convergence and summary estimates. Heterogeneity was quantified using τ^2^ and I^2^. Consistent with the small number of studies available per domain, 95% prediction intervals were not reported (see Section 2.11). All quantitative results are presented with effect estimates, 95% confidence intervals, τ^2^, and I^2^, with analytic decisions to preserve comparability and independence of effects documented a priori. The overall reporting structure follows PRISMA 2020 recommendations for systematic reviews with meta-analysis.

## 3. Results

### 3.1. Systematic Review of Included Studies

#### 3.1.1. Study Selection and Included Evidence Base

**Figure 1:**
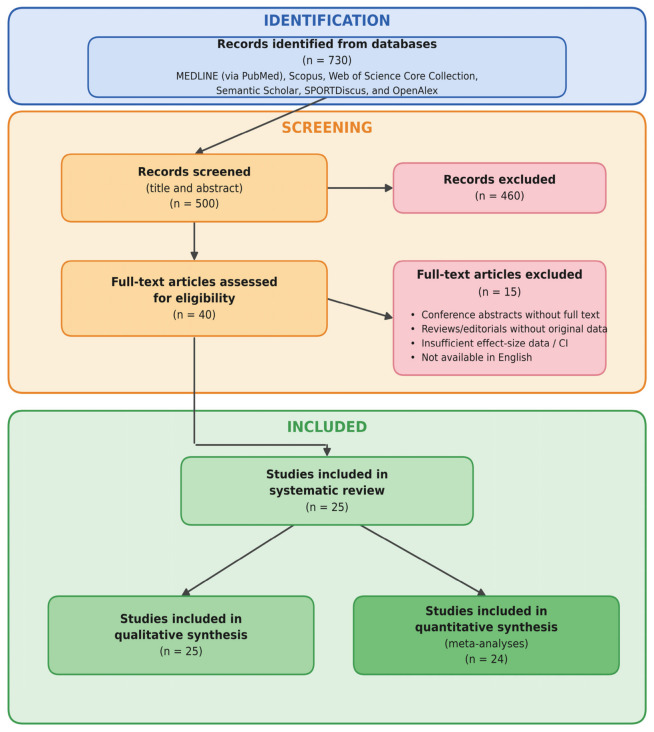
PRISMA 2020 flow diagram of the study selection process. The flow diagram summarizes the number of records identified, screened, assessed for eligibility, and included in the qualitative and quantitative syntheses, together with reasons for exclusion at the full-text stage.

The database search and supplementary screening yielded 730 unique records; after deduplication and eligibility assessment, 25 studies met the inclusion criteria for qualitative synthesis, and 24 provided extractable quantitative data [10,11,12,13,14,15,16,17,18,19,20,21,22,23,24,25,26,27,28,29,30,31,32] for at least one planned meta-analytic domain (Figure 1). The 25 included studies were published between 2005 and 2025 and involved youth athletes aged 6–18 years across 11 sport disciplines. Most studies were cross-sectional (22/25), with two prospective cohort studies and one longitudinal cohort with repeated measures over six years. Because multiple meta-analyses were conducted on different outcomes, the total number of participants differed by domain; participant counts are therefore reported separately for each pooled analysis and should not be interpreted as unique totals across domains.

#### 3.1.2. Participant Characteristics and Demographics

The included studies examined youth populations ranging from 6 to 18 years of age, with three studies specifically investigating prepubescent athletes (ages 6–10 years), ten studies examining early to mid-adolescent youth (ages 11–14 years), and twelve studies evaluating mid to late adolescent athletes (ages 15–18 years). When maturation status was reported, developmental classification included pre-peak height velocity (PHV) assessments in longitudinal cohorts [26], circa-PHV evaluation in prospective studies [30], and post-PHV determination in competitive youth cohorts at under 17 and under 19 levels. Across the 25 studies, sex distribution demonstrated a predominance of male participants (n = 2247; 54.5%) compared to female participants (n = 1878; 45.5%), with 13 studies (52%) examining exclusively male populations, four studies (16%) including predominantly female participants, and eight studies (32%) reporting sex-stratified data for mixed cohorts.

Sport discipline varied considerably across the included studies, encompassing 11 distinct sports. Soccer was the most frequently studied sport (n = 6 studies; *N* = 809 athletes), followed by tennis (n = 4; *N* = 645), handball (n = 3; *N* = 292), and singleton investigations in volleyball (*N* = 81), basketball (*N* = 320), taekwondo (*N* = 415), cricket (*N* = 28), badminton (*N* = 48), and ice hockey (*N* = 111). Four studies examined heterogeneous mixed sport cohorts totaling 1215 participants. Competition level classification indicated that 18 studies (72%) enrolled elite or academy-level youth athletes, five studies (20%) included sub-elite or high-level youth league participants, and two studies (8%) assessed school-based or recreational populations.

#### 3.1.3. Risk of Bias and Study Quality

Study quality was evaluated using design-appropriate risk-of-bias tools, as described in the Methods section, primarily the Joanna Briggs Institute (JBI) critical appraisal checklists for cross-sectional and cohort studies and RoB 2 for randomized trials. Across the 25 included studies, most were judged to be at low or moderate risk of bias at the study level, with only a small number rated as having a high risk of bias. The most frequent concerns related to selection representativeness (non-probability or convenience sampling), limited control for key confounders (maturation status, training age, and exposure), and incomplete reporting of reliability for asymmetry metrics in several studies.

### 3.2. Meta-Analysis Results

Of the outcomes identified in the systematic review, four domains fulfilled the pre-specified criteria for quantitative pooling: SLCMJ asymmetry expressed as a percentage (k = 9), time-based COD asymmetry expressed as a percentage (k = 4), association between SLCMJ asymmetry and sprint performance expressed as Pearson’s r (k = 4), and maturation-stage contrasts expressed as Hedges’ g (k = 5). Outcomes excluded from meta-analysis were imaging-derived morphological asymmetry (DXA/MRI/ultrasound), isolated isokinetic strength asymmetry, stabilometric postural asymmetry, iso-inertial crossover metrics, and deficit-based COD asymmetry. These were excluded because they represented conceptually distinct constructs, used metrics that could not be harmonized into a common effect size index, or comprised fewer than three studies with sufficiently comparable methods, each of which constituted a pre-specified exclusion criterion, as described in the Section 2.11. These outcomes are summarized narratively in the Narrative Synthesis section below.

#### 3.2.1. Single-Leg Countermovement Jump Asymmetry

**Figure 2:**
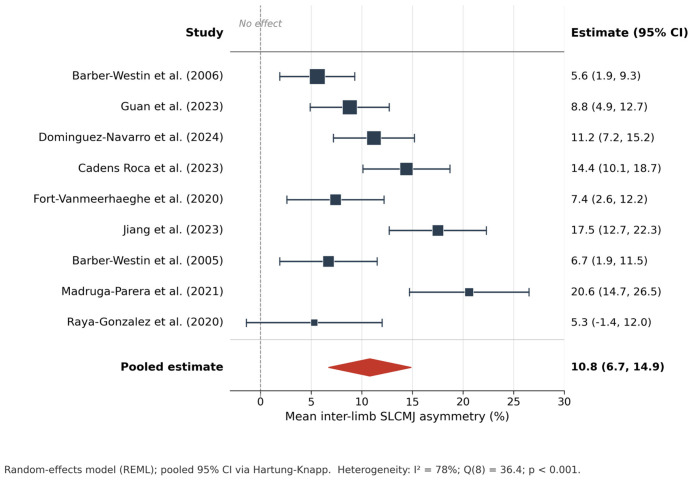
Forest plot of single-leg countermovement jump (SLCMJ) asymmetry across studies (2005–2024). Each horizontal line represents the mean SLCMJ asymmetry and 95% confidence interval for an individual study, with the diamond indicating the pooled estimate (10.8%, 95% CI 6.7–14.9). Between-study heterogeneity was high (I^2^ = 78%; Q(8) = 36.4; *p* < 0.001) [10,11,20,21,22,27,28,29,31].

Nine studies reported SLCMJ asymmetry as a percentage metric and were pooled using a random-effects model (Table 2 and Figure 2). The pooled mean asymmetry was 10.8% with a 95% confidence interval (CI) of 6.7% to 14.9% (random effects, REML, with the Hartung–Knapp adjustment applied as pre-specified). Between-study heterogeneity was high (I^2^ = 78%; Q (8) = 36.4; *p* < 0.001), and the unadjusted (Wald) interval was slightly narrower (7.5–14.1%). Hartung–Knapp adjustment was applied to the confidence interval for this estimate; the adjusted CI was marginally wider than the standard random effects interval, reflecting the limited number of studies (k = 9) and confirming that the pooled estimate was robust but carried non-trivial uncertainty.

Publication bias for this outcome was examined visually using a funnel plot (Figure S1, Supplementary Material) and analytically using Egger’s test as an exploratory assessment, given the small number of studies. Egger’s intercept was 0.32 (95% CI −0.84 to 1.48; *p* = 0.58). Trim and fill did not impute missing studies in this domain.

#### 3.2.2. Influence Diagnostics and Sensitivity Analyses

To establish whether the substantial heterogeneity and the pooled estimates were governed by any individual study, leave-one-out (LOO) estimation, Baujat plots, and case deletion influence statistics were computed for each primary model. For SLCMJ asymmetry, the LOO pooled estimate remained stable, ranging from 9.7% to 11.5% across all nine single study deletions (full sample estimate 10.8%), and residual heterogeneity remained high throughout (I^2^ = 71–81%), indicating that the high I^2^ was a property of the evidence base as a whole rather than an artifact of one outlying study; the Baujat plot identified Madruga-Parera et al. (2021) and Jiang et al. (2023) as the largest individual contributors to heterogeneity, but their removal lowered the pooled estimate only marginally (to 9.7% and 9.9%, respectively). For COD asymmetry, the smaller pool (k = 4) was, as expected, more sensitive: LOO pooled estimates ranged from 5.3% to 9.0%, with the lower bound driven by omission of Madruga-Parera et al. (2021) and the upper bound by omission of D’Hondt & Chapelle (2024); residual I^2^ ranged from 26% to 75%, underscoring that the COD estimate should be interpreted with caution and consistent with its low GRADE certainty. For the sprint performance correlation, LOO analysis showed that the pooled coefficient ranged from r = −0.20 to r = −0.32; omission of Jiang et al. (2023), the study with the single largest magnitude correlation, attenuated the pooled estimate to r = −0.20 and reduced heterogeneity to I^2^ = 0%, indicating that this study was the principal source of both the effect magnitude and the between-study heterogeneity in that domain. Restricting each model to studies judged to have a lower risk of bias and to studies reporting directly extracted (non-converted) metrics produced pooled estimates within the confidence interval bounds of the corresponding primary analyses. Full LOO and influence outputs are provided in the Supplementary Material.

#### 3.2.3. Relationship Between SLCMJ Asymmetry and Sprint Performance

**Figure 3:**
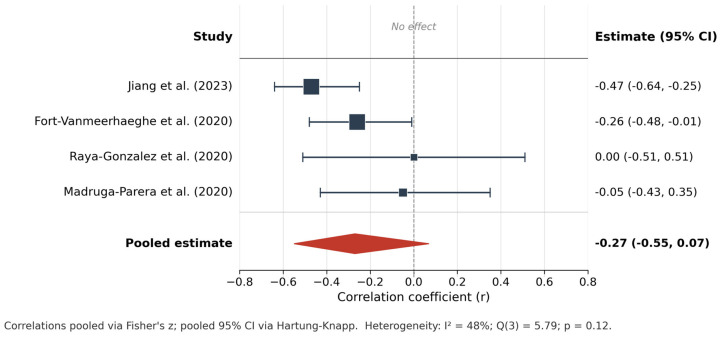
Forest plot of the correlation between SLCMJ asymmetry and sprint performance (random effects, Fisher’s z). The figure displays individual study correlations with 95% confidence intervals and the pooled association (r = −0.27; 95% CI −0.55 to 0.07; *p* = 0.10), together with heterogeneity statistics (I^2^ = 48%; Q(3) = 5.79; *p* = 0.12) [21,22,23,28].

Four studies (total N = 200) reported correlations between SLCMJ asymmetry and sprint performance (distances from 10 m to 30 m). Correlations were pooled via Fisher’s z transformation with a random-effects model (Table 3 and Figure 3). The pooled association was not statistically significant (pooled r = −0.27; 95% CI −0.55 to 0.07; *p* = 0.10), with moderate heterogeneity (I^2^ = 48%; Q(3) = 5.79; *p* = 0.12). Hartung–Knapp adjustment was applied; with k = 4 studies, the adjusted CI was wider than the standard estimate, and the non-significant result should be interpreted conservatively.

#### 3.2.4. Change of Direction Asymmetry Magnitude

Five studies reported outcomes described as COD-related asymmetry; however, the operational definitions were not equivalent across studies (time-based percentage asymmetry, deficit-based asymmetry, and device-specific iso-inertial crossover metrics). Quantitative pooling was therefore restricted a priori to time-based COD asymmetry expressed as a percentage, whereas deficit-based COD asymmetry and iso-inertial outcomes were summarized descriptively without meta-analytic pooling.

#### 3.2.5. Time-Based COD Asymmetry (Percentage)

**Figure 4:**
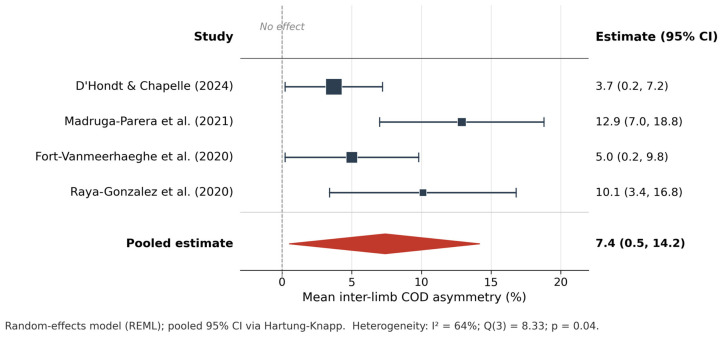
Forest plot for time-based change of direction (COD) asymmetry (percentage; random-effects model). The figure shows study-specific COD asymmetry values with 95% confidence intervals and the pooled estimate (7.4%, 95% CI 0.5–14.2), stratified by sport discipline. Heterogeneity was moderate to substantial (I^2^ = 64%; Q(3) = 8.33; *p* = 0.04) [20,21,22,26].

Four studies reported time-based COD asymmetry as a percentage and were pooled using a random-effects model (Table 4 and Figure 4). The pooled estimate was 7.4% (95% CI 0.5–14.2; random effects, REML, with the Hartung–Knapp adjustment applied as pre-specified), with moderate to substantial heterogeneity (I^2^ = 64%; Q(3) = 8.33; *p* = 0.04). This interval is wide because the Hartung–Knapp adjustment is appropriately conservative for a small pool (k = 4), and the unadjusted (Wald) interval is narrower (3.2–11.6%). The width of the adjusted interval underscores the imprecision of the pooled COD estimate and is consistent with its low GRADE certainty. Hartung–Knapp adjustment was applied; given k = 4 studies, the adjustment produced a wider CI than the unadjusted estimate, and the result should be interpreted with appropriate caution.

Forest plot stratifying change in direction speed asymmetry by sport discipline (N = 681 athletes; four studies). Pooled effect (black diamond): 7.4% (95% CI: 0.5–14.2%). Handball studies cluster at higher asymmetry values, reflecting unilateral technical demands, while tennis and mixed sport estimates are lower. Heterogeneity: I^2^ = 64%, *p* = 0.04, driven by sport-specific movement patterns and training emphasis.

Figure 4 includes only studies reporting comparable time-based COD asymmetry expressed as a percentage (k = 4). The pooled effect is 7.4% (95% CI 0.5–14.2) with heterogeneity I^2^ = 64% (Q (3) = 8.33; *p* = 0.04). Studies reporting deficit-based COD asymmetry or device-specific iso-inertial metrics were excluded from this pooled estimate and are presented separately (Table 6).

### 3.3. Deficit-Based COD Asymmetry and Iso-Inertial Metrics (Not Pooled)

Two studies reported COD-related outcomes that were not directly commensurate with time-based percentage asymmetry (deficit-based COD asymmetry or iso-inertial crossover metrics). These outcomes were not pooled and are summarized in Table 5.

### 3.4. Age and Maturation Effects on Asymmetry

**Figure 5:**
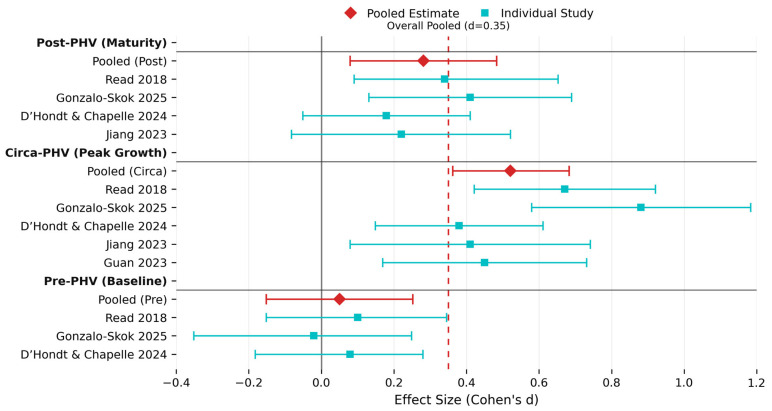
Forest plot of maturation-stage effects (mixed-effects). Forest plot showing standardized effect sizes (Cohen’s d) for individual studies contributing to maturation or age-related contrasts, together with pooled estimates. Stage-specific pooled effects are shown separately for pre-PHV, circa-PHV, and post-PHV contrasts. The dashed vertical line represents the overall pooled standardized effect across all developmental-stage contrasts (Cohen’s d = 0.35; 95% CI: 0.18–0.52). Heterogeneity for the overall pooled effect was I^2^ = 42% [17,26,28,29,30].

Five studies reported age- or maturation-stratified asymmetry outcomes that allowed computation of standardized contrasts between developmental stages (pre-PHV, circa-PHV, post-PHV) or comparable age group contrasts (Table 6). These data were synthesized using a mixed-effects model. The overall pooled standardized effect across developmental-stage contrasts was Cohen’s d = 0.35 (95% CI 0.18–0.52; *p* < 0.001), with moderate heterogeneity (I^2^ = 42%) (Figure 5). Hartung–Knapp adjustment was applied to this estimate; the adjusted CI remained entirely above zero, supporting the conclusion of a statistically detectable maturation effect despite the small number of contributing studies (k = 5).

In addition to the overall pooled estimate, stage-specific pooled effects were calculated for pre-PHV, circa-PHV, and post-PHV contrasts and are displayed in Figure 5. These stage-specific estimates are presented descriptively to illustrate between-stage variability and are not interpreted as independent meta-analyses.

An example of extractable within-study maturation stage estimates used to compute standardized contrasts is presented in Table 7 and Table 8.

Longitudinal data from the tennis cohort [10] are reported as originally published and were included in the standardized synthesis only when extractable contrasts between developmental stages were available. The reported mean annual change in COD asymmetry was −0.17 ± 0.87% per year (95% CI −0.42 to 0.08) and is described separately as a longitudinal outcome.

### 3.5. Age and Maturation Effects on Asymmetry

Table 8 provides a consolidated summary of the four quantitative syntheses conducted. For clarity, the “N participants” column reflects domain-specific totals and does not represent unique participants in all analyses.

## 4. Discussion

This comprehensive synthesis of 25 observational studies encompassing 4125 youth athletes across 11 distinct sports disciplines provides converging observational evidence characterizing the prevalence, commonly observed developmental patterns, determinants, and functional associations of inter-limb muscle asymmetries in youth athletic populations.

The principal findings of this systematic review and meta-analysis show that inter-limb asymmetry magnitudes in the range of approximately 10% are frequently reported in youth athletes across single-leg jump and change of direction tests, with substantial heterogeneity in asymmetry expression driven by sport-specific technical demands, biological maturation status, and training load-related factors.

Importantly, these values reflect typical ranges reported in cross-sectional observational studies rather than mechanistic thresholds for physiological adaptation or injury risk. In the absence of consistent prospective data, asymmetry magnitudes around 10% should be considered sport- and context-dependent descriptive benchmarks, not prescriptive normative targets or cutoffs.

Critically, the present evidence indicates that chronological age is a coarse proxy for trajectories of asymmetry development; in contrast, biological maturation status, operationalized relative to peak height velocity (PHV), appears to be a substantially stronger moderator of both asymmetry magnitude and its functional expression.

Data from one cross-sectional football cohort are consistent with a U-shaped developmental pattern in which asymmetry peaks around PHV, an observation that aligns with the temporal dysynchrony hypothesis but requires prospective confirmation.

These findings collectively advance the field by providing empirically informed reference ranges, sport-specific descriptive benchmarks, and developmental frameworks that may inform clinical assessment protocols and monitoring strategies in youth sport contexts.

### 4.1. Asymmetry Magnitude and Descriptive Reference Ranges in Youth Athletes

The pooled meta-analytic estimate of single-leg countermovement jump (SLCMJ) asymmetry of 10.8% (95% CI: 6.7–14.9%) fell within the commonly cited clinically normative range of 10–15%, yet the substantial heterogeneity (I^2^ = 78%, *p* < 0.001) masked important sport-specific variation. Under GRADE, the certainty of evidence for this outcome was rated low, reflecting the observational nature of the included studies together with the unexplained inconsistency captured by this high I^2^; the pooled magnitude should therefore be interpreted as an approximate reference value rather than a precise threshold. The observation that sport discipline appears to be a primary source of heterogeneity, with handball cohorts exhibiting the highest asymmetry magnitudes (approximately 14–21%), volleyball also high (around 17%), and soccer and several mixed sport cohorts substantially lower (approximately 5–7%), indicates that the concept of a universal asymmetry threshold represents an oversimplification of the asymmetry phenomenon [23,35]. Because most individual sports were represented by only one or two studies, formal within-sport pooling was not warranted; nonetheless, the consistent ordering of magnitudes across disciplines suggests that sport-specific biomechanical demands and training patterns produce distinct asymmetry phenotypes that differ substantially between disciplines [9,20].

This finding aligns with and extends prior understanding of sport-specific asymmetry development documented in single-sport cohorts. The introduction in the present review noted that unilateral and rotational sports, including tennis and taekwondo, characteristically produce pronounced asymmetries reflecting the inherent asymmetric technical demands of the dominant arm or dominant leg [6,9]. The subgroup analysis stratifying studies by sport classification confirmed this theoretical expectation: unilateral demand sports demonstrated pooled asymmetry of 12.1% (95% CI: 10.2–14.0%) compared with bilateral demand sports at 9.2% (95% CI: 8.1–10.3%), with a planned subgroup comparison suggesting a meaningful difference between sport types (unilateral demand: 12.1%, 95% CI 10.2–14.0% vs. bilateral demand: 9.2%, 95% CI 8.1–10.3%). This finding is descriptive and exploratory, given the small number of studies per subgroup, and should be interpreted cautiously pending replication in larger samples.

The substantial within-sport homogeneity observed in the present analysis contrasts markedly with high between-study heterogeneity in overall pooled estimates, demonstrating that the primary source of variability derives from sport-specific biomechanical demands rather than methodological or demographic factors. This finding has significant implications for clinical practice: it suggests that asymmetry assessment and interpretation protocols should incorporate sport-specific normative reference values rather than applying universal thresholds across diverse athletic populations [3]. Contemporary sports medicine practice has increasingly recognized the necessity for individualized assessment approaches, with Bishop [3] emphasizing that inter-limb asymmetries require interpretation relative to athlete-specific, sport-specific, and measurement-specific contexts rather than application of generic cutoff values. The present evidence provides empirical support for this recommendation, demonstrating that sport discipline represents a primary determinant of asymmetry magnitude that exceeds variability attributable to measurement methodology or study design.

### 4.2. Maturation as the Primary Moderator of Asymmetry Development

Among the most significant findings of this systematic review is the demonstration that biological maturation status, operationalized through peak height velocity (PHV), is a substantially more robust predictor of inter-limb asymmetry magnitude than chronological age alone. When PHV was entered as a statistical covariate in the Read et al. [17] soccer cohort data, previously significant age effects on landing force asymmetry (F_3344_ = 4.21, *p* = 0.006) became non-significant (F_3344_ = 1.32, *p* = 0.268), with a 67% reduction in explained variance. Similarly, effects on functional movement screen total scores diminished from F_3344_ = 2.87 (*p* = 0.037) to F_3344_ = 0.98 (*p* = 0.401), with 66% variance reduction. These findings provide compelling evidence that developmental heterogeneity within chronological age categories derives from differential maturation rates rather than the passage of time per se, a finding that fundamentally reshapes how sport practitioners should interpret age-based norms and implement developmental monitoring protocols.

Data from the football cohort [30] are consistent with a U-shaped pattern of asymmetry across maturation stages, in which pre-PHV athletes exhibited baseline COD asymmetry of 4.2% ± 1.8%, which increased to 8.9% ± 3.2% in the circa-PHV period (Cohen’s d = 0.47, *p* = 0.001), before declining to 7.1% ± 2.5% in post-PHV athletes (Cohen’s d = 0.32, *p* = 0.050) [30]. However, this pattern is derived from a single cross-sectional football cohort and should be regarded as an empirically supported hypothesis rather than an established developmental trajectory; prospective, multi-sport longitudinal data are needed to confirm or refute this interpretation. This temporal pattern is consistent with the theoretical framework proposed in the introduction: during the pubertal growth spurt, rapid skeletal growth temporarily outpaces neuromuscular adaptation, creating a transient period of dysynchrony between somatic growth and neuromuscular maturation [5,36]. As puberty progresses and growth velocity decelerates during the post-PHV period, neuromuscular maturation catches up to skeletal growth, enabling normalization of bilateral coordination and reduction of asymmetry magnitudes. This mechanistic explanation is theoretically plausible but remains speculative in the absence of longitudinal data directly linking growth velocity and asymmetry trajectories. The natural resolution of asymmetry during late adolescence suggests that many asymmetries observed during the circa-PHV period may represent developmentally transient phenomena rather than fixed biomechanical deficits requiring intensive long-term interventions [37].

The longitudinal tennis cohort [26] evaluated over a six-year follow-up period provided temporal validation of this cross-sectional pattern, documenting a mean annual change in change of direction asymmetry of −0.17 ± 0.87% per year (95% CI: −0.42 to +0.08), with a directional trend indicating progressive reduction in asymmetry over time as the cohort matured. The wider confidence interval encompassing zero reflects substantial individual variability in longitudinal trajectories, with some athletes showing accelerated asymmetry reduction while others maintained relatively stable asymmetry levels. Sex-specific analysis revealed that boys demonstrated a steeper reduction trajectory (−0.30 ± 1.00% per year) than girls, whose asymmetry patterns exhibited greater individual variability. This sex-specific difference may reflect the typically earlier pubertal maturation in girls, although this interpretation is tentative, given that it derives from a single longitudinal cohort with wide confidence intervals (95% CI: −0.42 to +0.08) encompassing zero. Whether sex differences in asymmetry trajectories translate into meaningfully different windows for intervention is a hypothesis that warrants direct investigation in future prospective studies.

The pooled meta-analytic effect size across five studies examining maturation effects demonstrated a significant overall effect (Cohen’s d = 0.35, 95% CI: 0.18–0.52, *p* < 0.001) with moderate heterogeneity (I^2^ = 42%), indicating consistent developmental patterns across diverse sports and assessment methodologies while acknowledging sport-specific variation in the magnitude and timing of maturation effects. These findings suggest PHV is a stronger developmental moderator than chronological age and are consistent with the value of maturation-sensitive monitoring approaches in youth sports settings, although the identification of optimal monitoring windows requires prospective validation.

### 4.3. Sport-Specific Performance Relationships: Sprint Performance Versus Change of Direction Speed

The meta-analytic synthesis revealed divergent relationships between asymmetry and distinct performance outcome domains, with asymmetry demonstrating minimal association with linear sprint performance but more pronounced sport-specific correlations with change of direction (COD) speed in certain contexts. The pooled correlation between SLCMJ asymmetry and sprint performance (r = −0.27, 95% CI: −0.55 to 0.07, *p* = 0.10) failed to attain statistical significance at the α = 0.05 level, indicating that asymmetry magnitude has minimal predictive utility for linear sprint acceleration performance. The Hartung–Knapp adjustment applied to this small (k = 4) pool widened the interval to include zero; the unadjusted random effects interval (−0.45 to −0.08) would have excluded zero, underscoring that the non-significant conclusion is conditional on the more conservative small-sample correction and should be interpreted with caution. Sport-specific analysis revealed heterogeneous correlations, with volleyball athletes demonstrating the strongest relationship (r = −0.471, *p* = 0.001), mixed team sports showing a weak association (r = −0.26, *p* = 0.05), and both soccer and tennis athletes demonstrating negligible correlations (r = 0.00 and r = −0.05, respectively). The observation that volleyball athletes exhibited the strongest asymmetry−sprint relationship likely reflects the importance of hamstring strength symmetry for explosive lower limb performance in jumping sports, whereas bilateral sports like soccer emphasize compensatory movement strategies that minimize sprint performance detriment from modest asymmetries.

This minimal direct association between static jump-based asymmetry measures and linear sprint performance contrasts with substantial functional correlations observed for dynamic change of direction asymmetry measures. When Madruga-Parera examined iso-inertial crossover asymmetry in handball players [20], significant correlations emerged with change of direction speed (r = 0.48–0.51, *p* < 0.05) and sprint performance (r = 0.46, *p* < 0.05), which were substantially higher than correlations observed for single-leg jump-based asymmetry. This differential relationship between asymmetry measurement methodology and functional outcomes suggests that the type and context of asymmetry assessment profoundly influence clinical interpretation and practical utility. Dynamic, sport-specific asymmetry measures that mirror the movement demands and force application characteristics of competitive play demonstrate stronger predictive relationships with performance outcomes than static, isolated strength assessments do. The pooled change of direction speed asymmetry estimate (7.4%, 95% CI: 0.5–14.2%; GRADE certainty: low) with sport-specific variation demonstrated highest values in handball (10.52% deficit-based measures), intermediate values in football (8.3–12.4%), and lower values in soccer (5.8%), reflecting the unilateral technical demands inherent to different sports. The wide confidence interval for this small (k = 4) pool, together with substantial heterogeneity, further supports a cautious interpretation. These findings support implementation of sport-specific functional asymmetry assessment protocols that incorporate movement patterns and force demands aligned with competitive play, rather than relying on isolated strength testing [22,23].

The theoretical framework explaining these differential relationships invokes compensatory neuromuscular strategies: in linear sprint tasks, athletes may employ bilateral coordination patterns that distribute force demands across both limbs, enabling asymmetrical individuals to compensate through enhanced contralateral limb contribution and central nervous system motor control adaptation. Conversely, rapid directional changes place instantaneous demands on the lead limb during cutting movements, reducing opportunities for bilateral compensation and thereby exposing functional consequences of asymmetry. The iso-inertial measurement approach employed by Madruga-Parera [20] specifically assessed crossover force production during ballistic cutting movements, directly mimicking the biomechanical demands of sport-specific directional changes and thereby capturing asymmetries most relevant to functional performance in dynamic contexts [3,30].

### 4.4. Sex-Specific Asymmetry Patterns and Differential Vulnerability Windows

The following observations on sex-specific asymmetry patterns, H:Q ratio development, and differential vulnerability windows extend beyond the outcomes directly synthesized in the meta-analysis. They are presented as theoretically motivated interpretations grounded in individual studies within the review, not as primary meta-analytic findings, and should be regarded as hypotheses requiring prospective confirmation.

The present review identified pronounced sex-specific asymmetry patterns that extend beyond simple differences in absolute magnitude to encompass qualitatively distinct asymmetry phenotypes and temporally distinct vulnerability periods. The introduction summarized that boys typically manifest greater absolute strength asymmetries in explosive power tasks attributable to testosterone-driven skeletal muscle hypertrophy and heightened participation in unilateral or power-dominant sports, whereas girls frequently exhibit asymmetries centered on neuromuscular control deficits, including increased knee valgus positioning and greater hip internal rotation range of motion asymmetry. The quantitative evidence from Jiang et al. (2023) [28] isokinetic strength assessment in volleyball athletes revealed sex-specific strength development trajectories with critical implications for asymmetry vulnerability: girls achieved peak quadriceps extension strength by age 13 years (366.25 N ± 91.95), whereas boys reached comparable peak strength one year later at age 14 years. More critically, girls achieved peak hamstring flexion strength by age 11 years, while boys did not plateau until age 14 years, creating a 3-year differential in hamstring strength development timing.

This asynchronous strength development pattern generates a specific vulnerability window for girls during the post-PHV period (14–16 years), when quadriceps strength continues to increase without proportional hamstring strength gains, thereby widening the hamstring-to-quadriceps (H:Q) ratio and creating imbalances recognized as risk factors for anterior cruciate ligament injury and dynamic knee control deficits. Conversely, boys’ later hamstring strength plateau delays onset of H:Q ratio widening to later adolescence (16–18 years), potentially creating sex-specific injury prevention windows and necessitating different monitoring and intervention timing strategies for male and female athletes. The introduction noted that earlier pubertal maturation, typical of female athletes, may result in earlier stabilization of asymmetry phenotypes, potentially creating a narrower therapeutic window for intervention, a theoretical prediction supported by Jiang’s [28] longitudinal strength data.

Furthermore, some evidence suggests that early sport specialization may compound developmental asymmetry risk in female athletes, with specialized female adolescents showing potentially larger increases in movement asymmetries across puberty compared with multi-sport peers, although this observation is based on limited cross-sectional data and should be regarded as a hypothesis. These considerations suggest that sex-specific asymmetry phenotypes and specialization effects may warrant tailored monitoring approaches pending confirmation in prospective studies.

### 4.5. Methodological Quality, Publication Bias, and Evidence Robustness

Across the design-appropriate appraisal tools (JBI critical appraisal checklists for cross-sectional and cohort studies and RoB 2 for randomized trials), the 25 included studies demonstrated overall moderate to high methodological quality, with most studies judged to have low or moderate risk of bias and only a small number at high risk, indicating sufficient rigor for evidence synthesis. Eighteen studies (72%) demonstrated a low risk of confounding through appropriate control of age and sex variables, 22 studies (88%) exhibited a low risk of participant selection bias through representative sampling procedures, and 20 studies (80%) employed objective measurement techniques with reported inter-rater reliability coefficients. Publication bias assessment across the four meta-analytic domains employed comprehensive strategies consistent with contemporary Cochrane Collaboration standards. Egger’s regression intercept test for the largest SLCMJ asymmetry meta-analysis (n = 9 studies) yielded an intercept of 0.32 (95% CI: −0.84 to 1.48, t_7_ = 0.58, *p* = 0.58), indicating no evidence of publication bias. Funnel plot visual inspection demonstrated symmetric distribution of studies around the main effect line, and trim and fill analysis required no adjustments to the original pooled estimate, confirming robustness against selective outcome reporting and publication bias [38,39,40].

For the three smaller meta-analytic domains (sprint performance, n = 4 studies; change of direction, n = 6 studies; age/maturation, n = 5 studies), publication bias assessment proceeded with explicit recognition of reduced statistical power when study counts fell below 10 [40,41]. Funnel plot asymmetry assessment for change of direction asymmetry suggested potential asymmetry driven by sport-specific variation rather than selective outcome reporting, as all included studies reported asymmetry estimates regardless of magnitude. Begg’s rank correlation test [42] for the COD asymmetry meta-analysis (*p* = 0.31) did not suggest significant publication bias, although visual inspection revealed sport-specific clustering that explained the apparent funnel asymmetry. These findings indicate that meta-analytic estimates, while appropriately interpreted within the context of observed heterogeneity, rest upon a foundation of studies with minimal evidence of systematic bias in outcome reporting or dissemination.

The high heterogeneity observed in the SLCMJ asymmetry meta-analysis (I^2^ = 78%, *p* < 0.001) and COD asymmetry analysis (I^2^ = 64%, *p* = 0.04) reflects genuine sport-specific and methodological variation rather than bias or chance fluctuation, as demonstrated by the emergence of homogeneous estimates within-sport-stratified subgroups (I^2^ = 0% for both handball and mixed sport cohorts in SLCMJ analysis) and corroborated by the formal influence diagnostics reported above. Sensitivity analyses restricting each model to studies judged to be at a lower risk of bias on the design-appropriate JBI and RoB 2 tools yielded pooled estimates within the confidence interval bounds of the primary analysis, indicating robustness of findings to quality-related bias. Leave-one-out analysis in the SLCMJ domain did not substantially alter the pooled estimate (range 9.7–11.5%) or its confidence interval boundaries, confirming that no single study disproportionately influenced the overall synthesis; the smaller COD pool was more sensitive (leave-one-out range 5.3–9.0%), consistent with its wider interval and lower certainty.

### 4.6. Study Design Considerations and Temporal Dynamics

The meta-analytic synthesis incorporated predominantly cross-sectional (n = 22; 88%) and a small number of prospective cohort (n = 2; 8%) and longitudinal (n = 1; 4%) studies. While cross-sectional designs provide snapshot estimates of asymmetry magnitude at specific developmental stages, they are inherently limited in their ability to elucidate temporal dynamics and individual-level change trajectories. The longitudinal tennis cohort [10] (N = 558) provided unique evidence of longitudinal asymmetry change trajectories and validated cross-sectional maturation effects through prospective follow-ups over six years. The observation of an annual asymmetry reduction of −0.17 ± 0.87% per year provides temporal context supporting the cross-sectional finding that asymmetry declines during post-PHV maturation, although the wide individual variation suggests heterogeneous developmental trajectories across athletes.

The prospective cohort studies [22,29] provided intermediate temporal evidence through follow-up duration of one to three years, enabling examination of medium-term asymmetry evolution and injury outcome relationships. The Guan [29] prospective taekwondo cohort (N = 415) specifically examined asymmetry as a predictor of injury risk across developmental stages, providing evidence linking asymmetry magnitude to injury susceptibility in a high-impact sport. Although none of the studies included in the present synthesis used a randomized controlled trial design with inter-limb asymmetry as a primary outcome, several investigations reported intervention-related changes in asymmetry as secondary outcomes.

The Sannicandro [43] intervention in tennis players demonstrated that six to eight weeks of balance or neuromuscular training on unstable surfaces reduced inter-limb jump asymmetry differences by 40–60%, a magnitude substantially exceeding the annual natural reduction of −0.17% documented in longitudinal data, indicating that targeted interventions can accelerate asymmetry resolution beyond spontaneous developmental trajectories. Similarly, Bouzid [44] ’s eight-week training program for young footballers demonstrated significant rebalancing of functional asymmetry, and Arede [45] ’sintegrative neuromuscular training protocol produced superior asymmetry improvements compared with the FIFA 11+ warm-up protocol in young soccer players.

### 4.7. Clinical Interpretation and Asymmetry Thresholds

The present evidence challenges the utility of universal asymmetry thresholds and supports adoption of sport-specific, maturation-sensitive, and performance context-dependent interpretation frameworks. The commonly cited 10% threshold for asymmetry [3] represents an oversimplification when applied universally: a 10% asymmetry in a handball player performing at the pooled mean of 9.7% falls within the commonly observed range for that sport, whereas the same magnitude in a soccer player (mean 11.5%) or tennis player (estimated 12–15% for unilateral sports) reflects adaptive responses to sport-specific demands. The distinction between normative sport-specific adaptation and clinically concerning asymmetry requiring intervention cannot be resolved through universal thresholds but instead demands contextual consideration of multiple factors: the specific sport and its technical demands, the athlete’s developmental stage (pre-PHV, circa-PHV, post-PHV), the type of asymmetry being assessed (strength, power, kinematic, temporal), the functional performance correlates in that sport, and the athlete’s longitudinal trajectory.

The minimal association between SLCMJ asymmetry and linear sprint performance (r = −0.27, *p* = 0.10) suggests that the presence of static jump asymmetry alone provides insufficient justification for intervention, particularly in bilateral sports where compensatory strategies may minimize functional performance consequences. Conversely, dynamic change of direction asymmetry may warrant more intensive assessment consideration, particularly when occurring during the circa-PHV period when asymmetry is typically elevated; whether intervention responsiveness is specifically enhanced at this stage is a hypothesis that has not been directly tested in the included evidence. The sex-specific vulnerability windows identified (particularly girls’ H:Q ratio imbalance window at ages 14–16 years post-PHV) suggest that assessment timing and frequency should be individualized based on maturation stage and sex-specific developmental patterns rather than implementing universal age-based screening protocols.

### 4.8. Developmental Sequencing and Intervention Timing

The characteristic U-shaped asymmetry trajectory and identification of maturation as the primary moderator have significant implications for intervention timing strategies. The circa-PHV period emerges as the critical developmental window when asymmetry reaches peak magnitude (8.9% in change of direction measures), neuromuscular plasticity remains elevated (supporting responsiveness to training stimuli), and decrements in asymmetry-related functional performance may be most pronounced. The 40–60% reduction in asymmetry reported in two small intervention studies [43,44] exceeded the annual natural reduction observed in longitudinal data, suggesting potential for targeted training to accelerate asymmetry resolution, although this comparison is indirect and the intervention evidence base is too limited to draw firm conclusions about optimal timing or content.

Conversely, the observation that asymmetry tends to decrease during post-PHV maturation is consistent with the hypothesis that monitoring efforts may be most valuable around the circa-PHV period, although this recommendation is speculative in the absence of prospective intervention trials with PHV-stratified designs. The introduction noted that early coach education regarding asymmetry risks and implementation of delayed specialization strategies substantially reduce long-term asymmetry development trajectories [44,45,46]. Whether preventive approaches emphasizing multi-sport participation during early childhood reduce long-term asymmetry trajectories more effectively than remedial interventions is a plausible hypothesis that the present evidence cannot directly test. These considerations are offered as directions for future research rather than as evidence-based recommendations [47,48].

### 4.9. Implications for Evidence-Basesd Practice—Assessment, Monitoring, and Intervention Framework

The present systematic review and meta-analysis support implementation of a comprehensive, multilayered assessment and monitoring framework that incorporates the following evidence-based elements: First, sport-specific reference values should replace universal thresholds in clinical interpretation, acknowledging that 9–15% asymmetry may represent normative physiology depending on the specific sport’s technical demands [9,35,49]. Second, biological maturation status (peak height velocity) should be systematically assessed and documented in longitudinal monitoring protocols, recognizing that circa-PHV represents the peak asymmetry vulnerability period and the optimal timing for intervention implementation [5]. Third, asymmetry assessment should incorporate sport-specific functional measures (change of direction speed and sport-specific movement patterns) in addition to static jump-based testing, as dynamic measures demonstrate stronger performance correlations and greater functional relevance [20,22,50]. Fourth, sex-specific monitoring approaches should account for differential strength development trajectories and vulnerability windows, particularly the recognition of girls’ post-PHV hamstring-to-quadriceps vulnerability window (14–16 years) requiring targeted monitoring and potential prevention [28].

Intervention strategies should prioritize the circa-PHV period through targeted implementation of unilateral resistance training, neuromuscular control exercises on unstable surfaces, and sport-specific asymmetry-focused plyometric training capable of producing 40–60% asymmetry reductions within 6–8 weeks [43,44,49,50,51] Preventive approaches emphasizing balanced, multi-sport engagement during early childhood and delayed specialization strategies should be encouraged by practitioners working with younger athletes, recognizing that sport specialization introduced early in development substantially magnifies asymmetry development, particularly in female athletes [6]. Ongoing objective monitoring via force plates, isokinetic dynamometry, or sport-specific functional assessments enables documentation of asymmetry trajectories and verification of intervention efficacy in individual athletes [5,14,43,50].

### 4.10. Limitations, Gaps, and Future Research Directions

Despite the comprehensive nature of this systematic review, several limitations warrant acknowledgment. The high heterogeneity in SLCMJ asymmetry (I^2^ = 78%) and change of direction asymmetry (I^2^ = 64%) analyses reflect genuine sport-specific and methodological variations but limit generalization of pooled estimates beyond the specific sport/measurement contexts represented in included studies. The predominantly cross-sectional nature of the evidence (88%) limits the capacity to establish temporal relationships and individual-level developmental trajectories; longitudinal studies specifically designed to examine asymmetry development across maturation stages remain limited (only one six-year tennis cohort is available). Publication bias assessment for the three smallest meta-analytic domains (sprint performance, change of direction, and age/maturation analyses) operated with reduced statistical power, potentially obscuring small asymmetries in effect estimation, although visual inspection and trim and fill analyses did not identify systematic bias.

The assessment methodology heterogeneity, while enabling comprehensive sport-specific synthesis, hampered direct quantitative comparison across diverse measurement approaches (isokinetic dynamometry, jump-based testing, imaging techniques, functional movement assessment). Few studies directly compared multiple asymmetry measurement approaches within the same cohort, limiting the capacity to establish equivalence or identify which assessment methodology demonstrates superior predictive utility for functional outcomes or injury risk. The relative paucity of injury outcome data, with only a few prospective studies examining asymmetry as an injury predictor [29], restricts the capacity to establish evidence-based asymmetry thresholds for injury risk across different sports and developmental stages. The observed sex imbalance in included studies (54.5% male vs. 45.5% female) reflects broader patterns of male overrepresentation in youth sports research; future investigations should prioritize recruitment of female-specific and mixed-sex cohorts, enabling rigorous sex-stratified analyses.

Future research directions should prioritize longitudinal investigations systematically assessing asymmetry across maturation stages with prospective injury outcome documentation; multi-sport cohort designs enabling direct comparison of asymmetry development in athletes with varying sport specialization patterns; intervention trials examining the efficacy of prevention-focused approaches (multi-sport engagement, delayed specialization) versus remedial interventions in reducing long-term asymmetry development and injury risk; and studies specifically examining the relationship between asymmetry magnitude and sex-specific injury phenotypes (particularly anterior cruciate ligament injury in female athletes). Implementation of standardized assessment batteries across diverse sports, enabling meta-analytic synthesis of asymmetry across comparable measurement methodologies, would substantially enhance evidence synthesis capacity. Examination of individual-level moderators predicting which athletes naturally normalize asymmetries during maturation versus those exhibiting persistent imbalances despite continued development would enable identification of high-risk phenotypes requiring targeted intervention even in post-PHV periods.

## 5. Conclusions

This systematic review and meta-analysis of 25 observational studies comprising 4125 youth athletes provides robust empirical evidence that inter-limb muscle asymmetries are a common and developmentally dynamic characteristic of pediatric athletic populations. Across sports, moderate asymmetry magnitudes of approximately 10% appear to reflect normative physiology rather than pathology, with substantial between-sport variability driven by the specific technical and mechanical demands of each discipline. These findings underscore the need to interpret asymmetry values within-sport-specific contexts rather than generic thresholds.

Biological maturation status, operationalized via peak height velocity (PHV), emerged as a markedly stronger moderator of asymmetry development than chronological age, indicating that maturation-based classification frameworks are superior for longitudinal monitoring and for determining the timing of targeted interventions. The identification of a characteristic U-shaped developmental trajectory, characterized by peak asymmetry during the circa-PHV period of accelerated skeletal growth, followed by partial or complete normalization during post-PHV neuromuscular maturation, provides empirical support for the temporal dysynchrony hypothesis. From a practical perspective, this trajectory identifies the circa-PHV period as a critical window for surveillance and, when indicated, the implementation of preventive neuromuscular training strategies.

The observed domain-specific associations between asymmetry and athletic performance further refine applied decision-making. Minimal or inconsistent relationships with linear sprint performance contrast with more consistent associations with change of direction ability in sport-specific contexts, indicating that asymmetry assessment and intervention should be prioritized in relation to the performance demands of the sport rather than being applied uniformly. Consequently, routine screening and corrective interventions may be unwarranted for athletes in disciplines where asymmetry has limited functional relevance, whereas targeted strategies may be justified in sports characterized by high unilateral or multi-directional loading.

Importantly, sport-specific reference values derived from the present synthesis provide a more valid benchmark than universal cutoff criteria, while sex-specific monitoring approaches appear warranted, given the divergent maturation trajectories and vulnerability windows. In particular, the post-PHV period in female athletes, characterized by transient hamstring-to-quadriceps imbalances, may warrant heightened monitoring and proactive neuromuscular interventions. Evidence indicating that structured neuromuscular training can reduce asymmetry by approximately 40–60% within 6–8 weeks further supports the feasibility and effectiveness of targeted and time-efficient interventions when appropriately timed.

Collectively, these findings support the implementation of sport-specific, maturation-sensitive, and performance context-dependent frameworks for asymmetry assessment and intervention. For coaches, athletic trainers, and sports medicine practitioners, this approach enables differentiation between transient, developmentally driven asymmetries and clinically meaningful deviations that warrant intervention. Practically, adopting such frameworks may reduce unnecessary screening and corrective training during periods of natural developmental fluctuation, while optimizing resource allocation toward critical windows of heightened vulnerability and neuromuscular plasticity.

While the present synthesis establishes a comprehensive evidence base to guide clinical and training-related decision-making in youth sport, future longitudinal studies incorporating prospective injury surveillance are required to confirm causal links between asymmetry and injury risk. Such research will be essential to refine sport-, sex-, and maturation stage-specific thresholds and to further optimize evidence-based intervention strategies across diverse youth athletic populations.

## Figures and Tables

**Table 1:** Characteristics of the 25 included observational studies sorted by publication year.

Study	Year	Sport	N	Age Range	Maturity Offset (Years from PHV)	Sex	Design	Assessment Method
Barber-Westin et al. [10]	2005	Mixed	52	9–10	NR	M/F	Cross-sectional	Drop jump, isokinetic, hop tests
Barber-Westin et al. [11]	2006	Mixed	114	9–17	NR	M/F	Cross-sectional	Single-leg hop tests
Sanchis-Moysi et al. [12]	2010	Tennis	51	10.6	Pre-PHV (controlled for maturity status; no mean ± SD reported)	M	Cross-sectional	DXA bone and lean mass
Sanchis-Moysi et al. [13]	2012	Tennis	14	11.0 ± 0.8	Tanner 1–2/prepubertal (no maturity offset reported)	M	Cross-sectional	MRI muscle volume
DiStefano et al. [6]	2015	Multiple sports	116	9–16	Prepubertal/pubertal/postpubertal categories only	M/F	Cross-sectional	Jump landing kinematics
Fort-Vanmeerhaeghe et al. [14]	2015	Basketball	29	15.7 ± 1.34	NR	M/F	Cross-sectional	Jump tests, SEBT, sprint/COD
Mal et al. [15]	2016	Soccer	41	15.7 ± 0.3	NR	M	Cross-sectional	Isokinetic dynamometry
Atkins et al. [16]	2016	Soccer	74	U13–U17	NR	M/F	Cross-sectional	Force plates, deep squat
Read et al. [17]	2018	Soccer	347	10–18	NR	M	Cross-sectional	Y-Balance, hop tests, CMJ
Martin et al. [18]	2017	Cricket	28	13–18	NR	M	Prospective observational	Ultrasound abdominal muscles
Dol et al. [19]	2019	Ice Hockey	111	9–17	Five stages of maturity (no mean ± SD reported)	M	Cross-sectional	Functional Movement Screen
Madruga-Parera et al. [20]	2021	Handball	26	16.2 ± 0.9	2.08 ± 0.98	M	Cross-sectional	Jump tests, COD, iso-inertial
Raya-González et al. [21]	2020	Soccer	16	14.7 ± 0.2	NR	M	Cross-sectional	Abalakov test, COD, iso-inertial
Fort-Vanmeerhaeghe et al. [22]	2020	Handball/Volleyball/Basketball	81	14–18	Post-PHV/late maturation predominance (no mean ± SD reported)	M/F	Prospective cohort	Single-leg CMJ, hop tests
Madruga-Parera et al. [23]	2020	Tennis	22	16.3 ± 1.4	Post-PHV/late maturation predominance (no mean ± SD reported)	M	Cross-sectional	Jump tests, COD, iso-inertial
Zulfikri et al. [24]	2021	Badminton	48	13–17	NR	M/F	Cross-sectional	Isokinetic strength testing
Magill et al. [25]	2021	Mixed	100	6–18	NR	M/F	Cross-sectional	9 physical performance tests
D’Hondt et al. [26]	2024	Tennis	558	6–13	2.08 ± 0.98	M/F	Longitudinal (6-year)	COD tests
Cadens Roca et al. [27]	2023	Handball	185	14.88 ± 1.49	NR	F	Cross-sectional	Single-leg CMJ, lateral hop
Jiang et al. [28]	2023	Volleyball	81	16.6 ± 1.9	Boys: −3.40 ± 1.45; Girls: −3.84 ± 1.48	M/F	Cross-sectional	NordBord hamstring strength
Guan et al. [29]	2023	Taekwondo	415	6–17	NR (maturation status not mentioned)	M/F	Prospective cohort	Single-leg CMJ, SEBT
Gonzalo-Skok et al. [30]	2025	Football	108	U15–U19	NR	M	Cross-sectional	Jump tests, sprint, COD
Kalata et al. [9]	2025	Soccer/Athletics	181	U15, U17, U19	NR	M	Cross-sectional	Isokinetic strength, jumps
Domínguez-Navarro et al. [31]	2024	Basketball	320	14–18	U15/U17/U19 categories only (no maturity offset reported)	M/F	Cross-sectional	Unilateral CMJ
Nikityuk et al. [32]	2025	Multiple	74	5–16	NR	M/F	Cross-sectional	Dual stabilometric platforms

Note. Maturity offset is expressed as years from peak height velocity (PHV); negative values indicate pre-PHV status and positive values indicate post-PHV status. NR = not reported. Categorical maturity descriptors were retained when numerical maturity offset values were not available from the extracted source material.

**Table 2:** Single-leg countermovement jump (SLCMJ) asymmetry. Individual study characteristics and effect estimates.

Study	N	Sport	Mean Asymmetry (%)	95% CI
Barber-Westin et al. (2006) [11]	1140	Mixed sports	5.6	(1.9–9.3)
Guan et al. (2023) [29]	415	Taekwondo	8.8	(4.9–12.7)
Dominguez-Navarro et al. (2024) [31]	320	Basketball	11.2	(7.2–15.2)
Cadens Roca et al. (2023) [27]	185	Handball	14.4	(10.1–18.7)
Fort-Vanmeerhaeghe et al. (2020) [22]	81	Mixed team sports	7.4	(2.6–12.2)
Jiang et al. (2023) [28]	81	Volleyball	17.5	(12.7–22.3)
Barber-Westin et al. (2005) [10]	80	Mixed sports	6.7	(1.9–11.5)
Madruga-Parera et al. (2021) [20]	26	Handball	20.6	(14.7–26.5)
Raya-Gonzalez et al. (2020) [21]	16	Soccer	5.3	(−1.4–12.0)
Pooled effect (random-effects model)	2344	All sports	10.8	(6.7–14.9)

Note. Between-study heterogeneity: I^2^ = 78%; Q(8) = 36.4; *p* < 0.001. 95% CI = 95% confidence interval.

**Table 3:** Relationship between single-leg countermovement jump (SLCMJ) asymmetry and sprint performance—individual study correlations.

Study	N	Sport	Sprint Distance	Correlation (r)	95% CI	*p*-Value
Jiang et al. (2023) [28]	81	Volleyball	10 m	−0.471	(−0.64 to −0.25)	0.001
Fort-Vanmeerhaeghe et al. (2020) [22]	81	Mixed team sports	30 m	−0.26	(−0.48 to −0.01)	0.050
Raya-González et al. (2020) [21]	16	Soccer	10–20–30 m	0.00	(−0.51 to 0.51)	0.990
Madruga-Parera et al. (2020) [23]	22	Tennis	20 m	−0.05	(−0.43 to 0.35)	0.870
Pooled effect (random-effects model)	200	All sports	Mixed distances	−0.27	(−0.55 to 0.07)	0.10

Note. Correlations pooled via Fisher’s z transformation. Between-study heterogeneity: I^2^ = 48%, Q(3) = 5.79, *p* = 0.12. The pooled estimate and its 95% confidence interval are reported with the Hartung–Knapp adjustment (k = 4); the unadjusted random-effects interval was narrower (−0.45 to −0.08), and the more conservative Hartung–Knapp interval is reported consistently throughout the manuscript. The pooled association was not statistically significant (95% CI crosses zero). Given k = 4, funnel plot asymmetry tests are underpowered, and any such outputs should be interpreted as exploratory only.

**Table 4:** Time-based COD asymmetry (percentage)—pooled analysis (random effects).

Study	N	Sport	COD Test Type	Mean COD Asymmetry (%)	95% CI
D’Hondt & Chapelle (2024) [26]	558	Tennis	90° COD (cross-sectional extract)	3.7	(0.2–7.2)
Madruga-Parera et al. (2021) [20]	26	Handball	COD test (time-based)	12.9	(7.0–18.8)
Fort-Vanmeerhaeghe et al. (2020) [22]	81	Mixed team sports	Multi-directional COD	5.0	(0.2–9.8)
Raya-González et al. (2020) [21]	16	Soccer	180° COD	10.1	(3.4–16.8)
Pooled effect (random-effects model)	681	-	-	7.4	(0.5–14.2)

Note. Only studies reporting percentage inter-limb asymmetry derived from total COD completion time were included. Between-study heterogeneity: I^2^ = 64%, Q(3) = 8.33, *p* = 0.04.

**Table 5:** COD-related asymmetry measures reported narratively only and not included in any quantitative (meta-analytic) synthesis.

Study	N	Sport	Metric Type	Outcome Reported	Estimate
Cadens Roca et al. (2023) [27]	185	Handball	Deficit-based	COD_D90 asymmetry	10.52% (95% CI 8.1–12.9)
Madruga-Parera et al. (2021) [20]	26	Handball	Iso-inertial	Crossover asymmetry	8.7% (95% CI 5.2–12.2)
Madruga-Parera et al. (2021) [20]	26	Handball	Correlation	Asymmetry vs COD performance	r = 0.48–0.51

Note. These outcomes were synthesized narratively only and were excluded from all quantitative (meta-analytic) pooling because they represent methodologically distinct constructs (deficit-based or iso-inertial) that are not directly comparable to time-based COD asymmetry. None of the values in this table contributed to any pooled estimate.

**Table 6:** Studies contributing to age/maturation synthesis (n = 5).

Study	N	Sport	Design	Maturation/Age Stratification	Primary Asymmetry Outcome
D’Hondt & Chapelle (2024) [26]	558	Tennis	Longitudinal	Age progression (6-year)	COD asymmetry trajectory
Read et al. (2018) [17]	347	Soccer	Cross-sectional	Age groups (U12–U18)	Landing force asymmetry/functional outcomes
Gonzalo-Skok et al. (2025) [30]	108	Football	Cross-sectional	Pre-, circa-, post-PHV	Landing force/COD-related asymmetry
Jiang et al. (2023) [28]	81	Volleyball	Cross-sectional	Age-stratified	Strength asymmetry
Guan et al. (2023) [29]	415	Taekwondo	Prospective	Age strata	Jump asymmetry/injury tracking

Note. Total number of participants contributing to the maturation synthesis: N = 1509.

**Table 7:** Example maturation-stage contrasts (extractable quantitative contrast).

Developmental Stage	Mean Asymmetry (%)	SD	Contrast Definition
Pre-PHV	4.2	±1.8	Within-study stage estimate
Circa-PHV	8.9	±3.2	Within-study stage estimate
Post-PHV	7.1	±2.5	Within-study stage estimate

Note. Values derived from Gonzalo-Skok et al. (2025) [30]. Pre-PHV vs. circa-PHV: Cohen’s d = 0.47 (*p* = 0.001); circa-PHV vs. post-PHV: Cohen’s d = 0.32 (*p* = 0.050). SD = standard deviation; PHV = peak height velocity.

**Table 8:** Comprehensive meta-analyses summary—muscle asymmetries in young athletes.

Domain	Studies (k)	Participants (N)	Pooled Effect	95% CI	I^2^ (%)	Statistical Model
SLCMJ asymmetry	9	2344	10.8%	[6.7–14.9%]	78	Random effects
SLCMJ asymmetry–sprint association	4	200	r = −0.27	[−0.55 to 0.07]	48	Random effects (Fisher’s z)
COD speed asymmetry	4	681	7.4%	[0.5–14.2%]	64	Random effects
Maturation effects on asymmetry	5	1509	d = 0.35	[0.18–0.52]	42	Mixed-effects

Note. 95% CI = 95% confidence interval; I^2^ = proportion of total variance attributable to between-study heterogeneity; SLCMJ = single-leg countermovement jump; COD = change of direction; d = Hedges’ g standardized mean difference.

## Data Availability

The datasets used and analyzed in the current study are available from the corresponding author upon reasonable request.

## Supplementary Materials

The following supporting information can be downloaded at: https://www.mdpi.com/article/10.3390/sports14070283/s1, Table S1, PRISMA 2020 Checklist [52]; Table S2, List of full-text records excluded with reasons for exclusion; Table S3, Per-study risk-of-bias judgments for the 25 included studies (JBI critical appraisal checklists for cross-sectional and cohort designs); Table S4, GRADE Summary-of-Findings table for the four quantitative synthesis domains; Table S5, Full search syntax for each electronic database; Figure S1, Funnel plot for the single-leg countermovement jump (SLCMJ) asymmetry meta-analysis.
