# Supplementary material for: Inter-Limb Muscle Asymmetries in Youth Athletes: A Comprehensive Systematic Review and Meta-Analysis of Single-Leg Jump and Change of Direction Speed Outcomes
Source: Sports (Basel). 2026 Jul 6;14(7):283. doi: 10.3390/sports14070283 (PMC13418687; doi:10.3390/sports14070283)
Supplement: Supplementary file 1 [file sports-14-00283-s001.zip › sports-4328509-supplementary.pdf]

Supplementary Materials

Contents

- Table S1. PRISMA 2020 Checklist
- Table S2. List of full-text records excluded with reasons for exclusion
- Table S3. Per-study risk-of-bias judgements for the 25 included studies (JBI critical appraisal checklists)
- Table S4. GRADE Summary of Findings table for the four quantitative synthesis domains
- Table S5. Full search syntax for each electronic database
- Figure S1. Funnel plot for the single-leg countermovement jump (SLCMJ) asymmetry meta-analysis

Table S1. PRISMA 2020 Checklist

Section and Topic	Item #	Checklist item	Location where item is reported
**TITLE**			
**Title**	**1**	Identify the report as a systematic review.	Inter limb Muscle Asymmetries in Youth Athletes: A Systematic Review and Meta-Analysis of Sport Specific Patterns, Maturation Effects and Performance Implications
**ABSTRACT**			
**Abstract**	**2**	See the PRISMA 2020 for Abstracts checklist.	Abstract (p. 3). The abstract reports background, objective, eligibility (PRISMA 2020, PROSPERO registration), information sources (six databases, search through October 2025), included studies (N = 25; 4,125 participants), synthesis methods (random-effects meta-analyses with I^2, τ^2), main pooled results with 95% CIs for SLCMJ, COD, sprint-asymmetry association, and maturation effects, and overall interpretation including limitations (lack of prospective injury data).
**INTRODUCTION**			
**Rationale**	**3**	Describe the rationale for the review in the context of existing knowledge.	Introduction, paragraphs 1–4 (pp. 3–4). Describes the construct of inter-limb asymmetry, the developmental complication of biological maturation/PHV in youth, methodological heterogeneity across the literature, and sport-specific phenotypes.
**Objectives**	**4**	Provide an explicit statement of the objective(s) or question(s) the review addresses.	Introduction, final two paragraphs (p. 4). Four primary aims (i–iv) and secondary aim are stated explicitly: (i) SLCMJ asymmetry magnitude, (ii) COD asymmetry magnitude, (iii) asymmetry sprint association, (iv) maturation/PHV modification.
**METHODS**			
**Eligibility criteria**	**5**	Specify the inclusion and exclusion criteria for the review and how studies were grouped for the syntheses.	Methods, "Eligibility Criteria" (p. 5). Inclusion (PICO + designs + language), exclusion (clinical populations, non-peer-reviewed, case reports/series < 10, validation-only studies, para-athletes, duplicate cohorts) and grouping into four pre-specified pooled domains plus narrative-synthesis outcomes.
**Information sources**	**6**	Specify all databases, registers, websites, organizations, reference lists and other sources searched or consulted to identify studies. Specify the date when each source was last searched or consulted.	Methods, "Information Sources and Search Strategy" (p. 5). Six databases (MEDLINE via PubMed, Scopus, Web of Science Core Collection, Semantic Scholar, SPORTDiscus, OpenAlex) searched from inception to October 2025; reference lists of included studies and relevant reviews screened manually; preprint servers and grey-literature sources also consulted.

Section and Topic	Item #	Checklist item	Location where item is reported
**Search strategy**	**7**	Present the full search strategies for all databases, registers and websites, including any filters and limits used.	Methods, "Information Sources and Search Strategy" (p. 5). High-level description of the search syntax (controlled vocabulary + free text terms across three blocks: asymmetry/laterality, youth, sport/performance). Full search syntax per database (six databases, with last searched dates and applied filters/limits) is provided as Supplementary Material, Table S5.
**Selection process**	**8**	Specify the methods used to decide whether a study met the inclusion criteria of the review, including how many reviewers screened each record and each report retrieved, whether they worked independently, and if applicable, details of automation tools used in the process.	Methods, "Study Selection Process" (p. 6). Two-stage dual-reviewer process (title/abstract then full text), independent screening, standardized eligibility form at full-text stage, reasons for exclusion recorded; no automation tools used.
**Data collection process**	**9**	Specify the methods used to collect data from reports, including how many reviewers collected data from each report, whether they worked independently, any processes for obtaining or confirming data from study investigators, and if applicable, details of automation tools used in the process.	Methods, "Data Extraction" (p. 6). Two reviewers extracted data independently using a piloted extraction template; disagreements resolved by consensus and, when needed, a third reviewer.
**Data items**	**10a**	List and define all outcomes for which data were sought. Specify whether all results that were compatible with each outcome domain in each study were sought (e.g. for all measures, time points, analyses), and if not, the methods used to decide which results to collect.	Methods, "Outcomes and operational definition of asymmetry" and "Data Extraction" (p. 6). Outcomes are: harmonized percentage between limb asymmetry for SLCMJ and time based COD; correlation between SLCMJ asymmetry and linear sprint performance; standardized contrasts in asymmetry by maturation stage. A pre specified hierarchy was used to choose one effect per study (e.g., sprint distance: 20 m > 10 m > 30 m).
	**10b**	List and define all other variables for which data were sought (e.g. participant and intervention characteristics, funding sources). Describe any assumptions made about any missing or unclear information.	Methods, "Data Extraction" (p. 6). Extracted variables: study identifiers, design, sport, competition level, sample size, sex, age, maturation status (incl. PHV), testing protocol, asymmetry computation method, reliability (ICC, CV). Missing dispersion values derived from available statistics (CIs, SE, t- or p-values); when derivation impossible, the study contributed only to narrative synthesis.
**Study risk of bias assessment**	**11**	Specify the methods used to assess risk of bias in the included studies, including details of the tool(s) used, how many reviewers assessed each study and whether they worked independently, and if applicable, details of automation tools used in the process.	Methods, "Risk of bias assessment" (p. 7). Design-appropriate tools: JBI critical appraisal checklist for analytical cross sectional studies; JBI cohort checklist for cohort studies; RoB 2 for randomized trials. Two reviewers independently; disagreements resolved by consensus. No automation tools.
**Effect measures**	**12**	Specify for each outcome the effect measure(s) (e.g. risk ratio, mean difference) used in the synthesis or presentation of results.	Methods, "Data synthesis and statistical analysis" (p. 7). Effect measures: pooled mean asymmetry (%) for SLCMJ and time-based COD; Pearson r (Fisher's z transformed) for asymmetry sprint association; standardized mean difference (Hedges' g / Cohen's d) for maturation-stage contrasts.
**Synthesis methods**	**13a**	Describe the processes used to decide which studies were eligible for each synthesis (e.g. tabulating the study intervention characteristics and comparing against the planned groups for each synthesis (item #5)).	Methods, "Eligibility Criteria" and "Outcomes and operational definition of asymmetry" (pp. 5–6). Pre-specified construct based grouping into four pooled domains; conceptually distinct outcomes (e.g., deficit-based COD, iso-inertial crossover, DXA/MRI, stabilometric) routed to narrative synthesis.
	**13b**	Describe any methods required to prepare the data for presentation or synthesis, such as handling of missing summary statistics, or data conversions.	Methods, "Outcomes and operational definition of asymmetry" and "Data Extraction" (p. 6). Harmonized metric: asymmetry (%) = 100 × |L − R| / max(L, R); LSI

Section and Topic	Item #	Checklist item	Location where item is reported
			converted to asymmetry as 100 − LSI; absolute magnitudes used when only directional asymmetry reported. Missing SD/SE derived from CIs, t-values or p-values via standard conversions.
	**13c**	Describe any methods used to tabulate or visually display results of individual studies and syntheses.	Methods, "Data synthesis and statistical analysis" (p. 7); Results - Tables 1–8 and Figures 1–5 (pp. 9–17). Forest plots for each pooled domain (Figures 2–5) and structured tables of individual-study estimates.
	**13d**	Describe any methods used to synthesize results and provide a rationale for the choice(s). If meta-analysis was performed, describe the model(s), method(s) to identify the presence and extent of statistical heterogeneity, and software package(s) used.	Methods, "Data synthesis and statistical analysis" (p. 7). Random-effects model as default (REML estimator of τ^2); Hartung–Knapp adjustment for CIs; prediction intervals for primary outcomes; heterogeneity quantified using Cochran's Q and I^2. Software: R (metaphor package) with parallel verification in Stata.
	**13e**	Describe any methods used to explore possible causes of heterogeneity among study results (e.g. subgroup analysis, meta-regression).	Methods, "Data synthesis and statistical analysis" (p. 7). Pre-specified subgroup analyses (sport-demand classification, sex, maturation stage, testing approach), conditional on a defensible minimum number of studies; meta-regression considered only where study counts allowed stable estimation.
	**13f**	Describe any sensitivity analyses conducted to assess robustness of the synthesized results.	Methods, "Data synthesis and statistical analysis" (p. 7). Leave-one-out diagnostics; restriction to studies at lower risk of bias; restriction to studies using directly reported (non-converted) asymmetry metrics.
**Reporting bias assessment**	**14**	Describe any methods used to assess risk of bias due to missing results in a synthesis (arising from reporting biases).	Methods, "Assessment of publication bias and small-study effects" (p. 8). Funnel plots and Egger's regression planned only where $k \geq 10$; for smaller pools, qualitative appraisal within GRADE for systematic effect-size–sample-size patterns and selective outcome reporting.
**Certainty assessment**	**15**	Describe any methods used to assess certainty (or confidence) in the body of evidence for an outcome.	Methods, "Certainty of evidence" (p. 7). GRADE framework with starting rating of "low" for observational evidence; downgrading for risk of bias, inconsistency, indirectness, imprecision, suspected publication bias; upgrading considered for large, consistent effects with dose response.
**RESULTS**			
**Study selection**	**16a**	Describe the results of the search and selection process, from the number of records identified in the search to the number of studies included in the review, ideally using a flow diagram.	Results, "Study Selection and included evidence base" (p. 8); PRISMA 2020 flow diagram in Figure 1 (p. 9). 730 unique records → 230 duplicates removed → 500 screened → 40 full-text assessed → 25 included in qualitative synthesis, 24 contributing to ≥1 meta-analytic domain.
	**16b**	Cite studies that might appear to meet the inclusion criteria, but which were excluded, and explain why they were excluded.	Results, p. 8 - full-text exclusion reasons are stated as recorded in the PRISMA flow diagram. The complete list of full text records excluded with the primary reason for exclusion is provided as Supplementary Material, Table S2.
**Study characteristics**	**17**	Cite each included study and present its characteristics.	Results, Table 1 (p. 10) - 25 included studies with year, sport, N, age range, sex, design and assessment method.
**Risk of bias in studies**	**18**	Present assessments of risk of bias for each included study.	Results, "Risk of bias and study quality" (p. 11) – body of evidence summary in main text, with per-study domain-level judgements provided as Supplementary Material. Table S3 reports JBI domain-level (D1–D8)

Section and Topic	Item #	Checklist item	Location where item is reported
			and overall Low/Moderate/High judgements for each of the 25 included studies; cohort items 9–11 are summarized in the Notes column.
**Results of individual studies**	**19**	For all outcomes, present, for each study: (a) summary statistics for each group (where appropriate) and (b) an effect estimate and its precision (e.g. confidence/credible interval), ideally using structured tables or plots.	Results -Table 2 (SLCMJ asymmetry, p. 11), Table 3 (asymmetry sprint correlations, p. 12), Table 4 (time-based COD asymmetry, p. 13), Table 5 (deficit based COD / iso-inertial outcomes, p. 15), Table 6 (maturation synthesis, p. 15), Table 7 (extractable maturation stage contrasts, p. 15). Each study presented with N, sport, point estimate and 95% CI.
**Results of syntheses**	**20a**	For each synthesis, briefly summarize the characteristics and risk of bias among contributing studies.	Results, narrative paragraphs preceding each pooled-domain table (pp. 11–17); contributing-study characteristics summarized in Tables 2–8.
	**20b**	Present results of all statistical syntheses conducted. If meta-analysis was done, present for each the summary estimate and its precision (e.g. confidence/credible interval) and measures of statistical heterogeneity. If comparing groups, describe the direction of the effect.	Results, pp. 11–17 and Table 8 (consolidated summary, p. 17): SLCMJ asymmetry 10.8% (95% CI 6.7–14.9; I^2 = 78%, k = 9); asymmetry sprint association r = −0.27 (95% CI −0.55 to 0.07; I^2 = 48%, k = 4); time-based COD asymmetry 7.4% (95% CI 0.5–14.2; I^2 = 64%, k = 4); maturation contrasts d = 0.35 (95% CI 0.18–0.52; I^2 = 42%, k = 5).
	**20c**	Present results of all investigations of possible causes of heterogeneity among study results.	Results and Discussion (pp. 14–22). Sport stratified subgroup analyses (handball, soccer, tennis, mixed cohorts) and unilateral vs bilateral demand sport classification reported in the Discussion; sport discipline identified as the dominant source of between study heterogeneity in SLCMJ and COD domains.
	**20d**	Present results of all sensitivity analyses conducted to assess the robustness of the synthesized results.	Discussion, "Methodological Quality, Publication Bias, and Evidence Robustness" (pp. 22–23). Leave-one-out and quality restricted sensitivity analyses reported as not materially altering pooled estimates or 95% CI boundaries.
**Reporting biases**	**21**	Present assessments of risk of bias due to missing results (arising from reporting biases) for each synthesis assessed.	Results, pp. 11–12 (Egger's intercept 0.32, 95% CI −0.84 to 1.48, p = 0.58 for SLCMJ; trim-and-fill not required); Discussion p. 22 (Begg's test for COD, p = 0.31). A funnel plot for the SLCMJ meta-analysis (k = 9), with the random effects pooled estimate, 95% pseudo-confidence envelope and Egger's regression annotation, is provided as Supplementary Material, Figure S1. For domains with k < 10 (sprint association, time-based COD, maturation contrasts) formal funnel-based testing was not undertaken and the qualitative judgement is reflected in the GRADE publication-bias domain (Table S4).
**Certainty of evidence**	**22**	Present assessments of certainty (or confidence) in the body of evidence for each outcome assessed.	Methods, "Certainty of evidence" (p. 7) — GRADE planned per outcome; per outcome certainty ratings are presented in the Supplementary Material. A GRADE Summary of Findings table for the four pre-specified pooled outcomes is provided as Supplementary Material, Table S4. Final certainty ratings: SLCMJ asymmetry magnitude - Very low; SLCMJ sprint association - Very low; time-based COD asymmetry - Very low; maturation stage contrast - Low. Downgrading reasons are given per outcome and per GRADE domain (risk of bias, inconsistency, indirectness, imprecision, publication bias).
**DISCUSSION**			

Section and Topic	Item #	Checklist item	Location where item is reported
**Discussion**	**23a**	Provide a general interpretation of the results in the context of other evidence.	Discussion, opening paragraphs and themed subsections (pp. 17–22): Asymmetry magnitude and normative physiology, Maturation as primary moderator, Sport specific performance relationships, Sex specific patterns.
	**23b**	Discuss any limitations of the evidence included in the review.	Discussion, “Limitations, Gaps, and Future Research Directions” (pp. 23–24). High heterogeneity, predominance of cross sectional designs, limited prospective injury data, methodological heterogeneity across asymmetry metrics, sex imbalance in included samples.
	**23c**	Discuss any limitations of the review processes used.	Discussion, “Limitations, Gaps, and Future Research Directions” (pp. 23–24). Reduced statistical power for publication-bias assessment in small pools (k < 10), inability to perform meta regression for small pools, restriction to English language full-text reports.
	**23d**	Discuss implications of the results for practice, policy, and future research.	Discussion, “Implications for Evidence-Based Practice - Assessment, Monitoring, and Intervention Framework” (pp. 24–25) and Conclusions (p. 25). Sport-specific reference values, maturation-based monitoring, prioritization of circa-PHV intervention windows, sex specific protocols, and research priorities (longitudinal designs with prospective injury surveillance).
**OTHER INFORMATION**			
**Registration and protocol**	**24a**	Provide registration information for the review, including register name and registration number, or state that the review was not registered.	Methods, “Study Design and Protocol Registration” (p. 5). Register: PROSPERO. Registration number: CRD420261294739.
	**24b**	Indicate where the review protocol can be accessed, or state that a protocol was not prepared.	Methods, p. 5. Protocol accessible via the PROSPERO record (https://www.crd.york.ac.uk/prospero/) under registration number CRD420261294739.
	**24c**	Describe and explain any amendments to information provided at registration or in the protocol.	Methods, “Study Design and Protocol Registration” and “Data synthesis and statistical analysis” (pp. 5, 7). Post-hoc restrictions introduced to prevent inappropriate pooling of heterogeneous outcomes (separation of time-based vs deficit-based COD; routing of iso-inertial and DXA/MRI/stabilometric outcomes to narrative synthesis) are explicitly described.
**Support**	**25**	Describe sources of financial or non-financial support for the review, and the role of the funders or sponsors in the review.	Funding statement (p. 26): “This research received no external funding.” No funder involvement to declare.
**Competing interests**	**26**	Declare any competing interests of review authors.	Conflicts of Interest statement (p. 26): “The authors declare no conflicts of interest.”
**Availability of data, code and other materials**	**27**	Report which of the following are publicly available and where they can be found: template data collection forms; data extracted from included studies; data used for all analyses; analytic code; any other materials used in the review.	Data Availability Statement (p. 26): “The datasets used and analyzed during the current study are available from the corresponding author upon reasonable request.”

From: Page MJ, McKenzie JE, Bossuyt PM, Boutron I, Hoffmann TC, Mulrow CD, et al. The PRISMA 2020 statement: an updated guideline for reporting systematic reviews. BMJ 2021;372:n71. doi: 10.1136/bmj.n71. Licensed under CC BY 4.0.

Table S2. List of full-text records excluded with reasons for exclusion

Table S2. Records assessed at the full text stage that did not meet the pre specified eligibility criteria, with the primary reason for exclusion. The fifteen entries below cover all reason categories that arose during full text adjudication: training intervention design without extractable observational baseline (k = 4); outcome construct outside scope, including upper limb or trunk asymmetry (k = 3); mixed adult, youth cohort with non-extractable youth subgroup data (k = 2); conference abstract / non-peer reviewed short report (k = 1); not available in English (k = 2; one Hungarian, one Portuguese); population outside scope (k = 1); outcome not eligible, injury incidence rather than asymmetry magnitude (k = 1); and sample size below the pre-specified minimum for pooling (k = 1). Reasons are categorized consistently with the eligibility criteria described in the Methods section and with the PRISMA 2020 reporting requirements. Records in this table are not cited in the synthesis but were screened and adjudicated by two reviewers independently.

#	Citation	DOI	Study design / sample	Reason for exclusion at full-text stage
1	Cools A, Johansson F, Cambier D, et al. (2010). Descriptive profile of scapulothoracic position, strength and flexibility variables in adolescent elite tennis players. Br J Sports Med.	10.1136/bjsm.2009.070128	Cross-sectional, 35 adolescent tennis players	Outcome construct outside scope: study quantified shoulder girdle (upper limb) position, strength and flexibility, not between limb lower limb asymmetry as defined a priori.
2	Sannicandro I. (2023). The effects of training with unstable surfaces on the strength and inter-limb asymmetry values in young soccer players. Med Sport.	10.23736/s0025-7826.22.04190-4	Longitudinal intervention study, 37 youth soccer players	Training-intervention design: primary aim was evaluation of an unstable surface training program; pre-intervention (baseline) asymmetry estimates not reported separately in a form compatible with the harmonized pooling metric.
3	Jackson SS, Sugimoto D, Howell D, Meehan W, Stracciolini A. (2016). Effects of Sports Specialization on the Rates of Iliotibial Band Syndrome in Pediatric Athletes. Med Sci Sports Exerc.	10.1249/01.mss.0000486526.79107.42	Cross-sectional questionnaire, 549 participants	Outcome not eligible: study quantified injury incidence (ITB syndrome) by specialization level, not between limb asymmetry as a measured construct.
4	Steidl-Müller L, Hildebrandt C, Müller E, et al. (2018). Limb symmetry index in competitive alpine ski racers: Reference values and injury risk identification. J Sport Health Sci.	10.1016/j.jshs.2018.09.002	Cross-sectional + longitudinal, 285 racers (incl. elite adults 20–34 y)	Mixed-age cohort included adult elite athletes (20–34 y); youth only subgroup data for the harmonized asymmetry metric were not separately extractable for pooling.
5	Moreno-Azze A, Arjol-Serrano JL, et al. (2021). Comparison of Three Eccentric Overload Training Strategies on Power Output and Interlimb Asymmetry in Youth Soccer Players. Int J Environ Res Public Health.	10.3390/ijerph18168270	10-week intervention, 35 male U-17 soccer players	Training-intervention RCT: comparison of three eccentric-overload protocols; pre-intervention baseline asymmetry not reported in a form compatible with the harmonized observational pooling metric.
6	Parpa K, Michaelides M. (2022). Anterior-Posterior and Inter-Limb Lower Body Strength Asymmetry in Soccer, Basketball, Futsal, and Volleyball Players. Medicina.	10.3390/medicina58081080	Cross-sectional, 254 athletes (adult + youth)	Mixed adult and youth cohort (adult mean ages 23.9–28.2 y; youth means 15.2–17.2 y); youth only estimates for the

#	Citation	DOI	Study design / sample	Reason for exclusion at full-text stage
				harmonized metric were not separately reported.
7	Arampatzis A, Frank J, Laube G, Mersmann F. (2019). Trunk muscle strength and lumbo-pelvic kinematics in adolescent athletes: Effects of age and sex. Scand J Med Sci Sports.	10.1111/sms.13503	Cross-sectional, 50 adolescent athletes	Outcome construct outside scope: study examined trunk muscle strength and lumbo-pelvic kinematics, not between limb asymmetry of the lower limbs.
8	Bouzid W, Rakaa OB, Chokri A, et al. (2025). Effect of an 8-week training program on the rebalancing of functional asymmetry in young footballers. Retos.	10.47197/retos.v70.117056	8-week intervention, 78 male youth footballers (U16–U18)	Training-intervention design; cited narratively in Discussion as evidence of intervention efficacy but not eligible for the observational asymmetry magnitude meta-analysis.
9	Peek K, Ford K, Myer G, Hewett T, Pappas E. (2020). Effect of maturation on knee extensor and flexor strength in male and female adolescent athletes. Br J Sports Med (IOC abstract).	10.1136/bjsports-2020-IOCAbstracts.321	Conference abstract, longitudinal cohort, 257 participants	Conference abstract / non-peer reviewed short report; insufficient methodological detail (specifically the asymmetry computation and dispersion statistics) for inclusion.
10	MacSweeney N, Shaw JW, Simkin GP, et al. (2024). Jumping Asymmetries and Risk of Injuries in Preprofessional Ballet. Am J Sports Med.	10.1177/03635465231218258	Cohort, 242 paraprofessional ballet dancers (11–19 y)	Population outside scope: paraprofessional ballet does not meet the pre specified definition of organized sport with competitive structure; sport classification framework not applicable.
11	Laczkó A, Gombos Z, Bogár L, Tóth L. (2022). Aszimmetriák felmérése és kiküszöbölése serdülőkorú kézilabdázó lányoknál. Acta Univ Eszterhazy Sport.	10.33040/actauniveszterhazysport.2024.53.7	Intervention study, 13 U15 female handball players	Not in English (Hungarian); also a training intervention design with n < 15 and limited dispersion reporting.
12	Ferreira JC, Araújo SR, Pimenta E, et al. (2018). Impacto do Nivel Competitivo e da Faixa Etaria Sobre Força e Assimetria de Futebolistas Jovens. Rev Bras Med Esporte.	10.1590/1517-869220184985	Cross-sectional, 151 male youth soccer players (U15–U20)	Not in English (Portuguese); cohort overlaps the upper boundary of the youth window (U20) without separately extractable youth only estimates.
13	Sannicandro I, Cofano GA, Rosa RA, Piccinno A. (2014). Balance training exercises decrease lower-limb strength asymmetry in young tennis players. J Sports Sci Med.	—	RCT intervention, 23 youth tennis players (mean 13 y)	Training-intervention RCT; cited narratively in Discussion as evidence of intervention efficacy. Pre-intervention baseline asymmetry not reported in a form compatible with the harmonized observational pooling metric.
14	Selmani A, Begu B. (2025). The Asymmetry Level of Hamstrings Strength in Female U16 Basketball Players. KOSALB Int J Hum Mov Sci.	10.70736/2958.8332.kosalb.53	Cross-sectional, 10 female U16 basketball players	Sample size below the pre-specified minimum (n = 10 < 15); single-modality isokinetic hamstring asymmetry

#	Citation	DOI	Study design / sample	Reason for exclusion at full-text stage
				contributes to narrative synthesis only and was not pooled.
15	Oyama S, Waldhelm A, Sosa A, Patel RR. (2017). Trunk Muscle Function Deficit in Youth Baseball Pitchers With Excessive Contralateral Trunk Tilt During Pitching. Clin J Sport Med.	10.1097/JSM.0000000000000396	Cross-sectional, 28 youth baseball pitchers	Outcome construct outside scope: study examined trunk muscle function and pitching kinematics, not between limb (lower limb) asymmetry as defined for the review.

Table S3. Per-study risk-of-bias judgements for the 25 included studies

Table S3. Per-study domain level risk of bias (RoB) judgements for the 25 studies included in the qualitative and quantitative syntheses. Cross sectional studies (CS) were appraised using the Joanna Briggs Institute (JBI) Critical Appraisal Checklist for Analytical Cross Sectional Studies (8 items); prospective cohort (PC) and longitudinal cohort (LC) studies were appraised using the JBI cohort checklist (11 items). Domain judgements were made independently by two reviewers and disagreements resolved by consensus; the overall RoB was derived from the pattern of unmet items.

#	Study	Sport	Design	JBI critical appraisal — domain-level judgements								Overall RoB	Notes
				D1	D2	D3	D4	D5	D6	D7	D8		
1	Barber-Westin et al. [10] (2005)	Mixed	CS	Y	Y	Y	Y	U	U	Y	Y	Moderate	—
2	Barber-Westin et al. [11] (2006)	Mixed	CS	Y	Y	Y	Y	U	U	Y	Y	Moderate	—
3	Sanchis-Moysi et al. [12] (2010)	Tennis	CS	Y	Y	Y	Y	Y	Y	Y	Y	Low	—
4	Sanchis-Moysi et al. [13] (2012)	Tennis	CS	Y	Y	Y	Y	U	U	Y	Y	Moderate	—
5	DiStefano et al. [6] (2015)	Multi-sport	CS	Y	Y	Y	Y	Y	Y	Y	Y	Low	—
6	Fort-Vanmeerhaeghe et al. [14] (2015)	Basketball	CS	Y	Y	Y	Y	U	U	Y	Y	Moderate	—
7	Malý et al. [15] (2016)	Soccer	CS	Y	Y	Y	Y	U	U	Y	Y	Moderate	—
8	Atkins et al. [16] (2016)	Soccer	CS	Y	Y	Y	Y	Y	U	Y	Y	Moderate	—
9	Read et al. [17] (2018)	Soccer	CS	Y	Y	Y	Y	Y	Y	Y	Y	Low	—
10	Martin et al. [18] (2017)	Cricket	PC	Y	Y	Y	Y	U	U	Y	Y	Moderate	*JBI cohort items 8–11: follow-up reported but loss-to-follow-up reasons not fully explored.*
11	Dol et al. [19] (2019)	Ice hockey	CS	Y	Y	U	Y	N	N	U	Y	High	*Grey-literature thesis; reliability of FMS scoring*

#	Study	Sport	Design	JBI critical appraisal — domain-level judgements								Overall RoB	Notes
				D1	D2	D3	D4	D5	D6	D7	D8		
													and confounder control not fully reported.
12	Madruga-Parera et al. [20] (2021)	Handball	CS	Y	Y	Y	Y	U	U	Y	Y	Moderate	—
13	Raya-González et al. [21] (2020)	Soccer	CS	Y	Y	Y	Y	U	U	Y	Y	Moderate	*Small sample (n=16); confounding by maturation not statistically adjusted.*
14	Fort-Vanmeerhaeghe et al. [22] (2020)	Team sports	PC	Y	Y	Y	Y	Y	Y	Y	Y	Low	*JBI cohort items 8–11: follow-up adequate and loss-to-follow-up handling described.*
15	Madruga-Parera et al. [23] (2020)	Tennis	CS	Y	Y	Y	Y	U	U	Y	Y	Moderate	*Small sample (n=22).*
16	Zulfikri et al. [24] (2021)	Badminton	CS	Y	Y	Y	Y	U	U	Y	Y	Moderate	—
17	Magill et al. [25] (2021)	Multi-sport	CS	Y	Y	Y	Y	Y	Y	Y	Y	Low	—
18	D'Hondt & Chapelle [26] (2024)	Tennis	LC	Y	Y	Y	Y	Y	Y	Y	Y	Low	*JBI cohort items 8–11: 6-year follow-up reported, loss-to-follow-up addressed.*
19	Cadens Roca et al. [27] (2023)	Handball	CS	Y	Y	Y	Y	Y	U	Y	Y	Moderate	—
20	Jiang et al. [28] (2023)	Volleyball	CS	Y	Y	Y	Y	Y	U	Y	Y	Moderate	—
21	Guan et al. [29] (2023)	Taekwondo	PC	Y	Y	Y	Y	Y	Y	Y	Y	Low	*JBI cohort items 8–11: prospective follow-up with injury monitorin*

#	Study	Sport	Design	JBI critical appraisal — domain-level judgements								Overall RoB	Notes
				D1	D2	D3	D4	D5	D6	D7	D8		
													g and complete outcome data.
22	Gonzalo-Skok & Bishop [30] (2025)	Football	CS	Y	Y	Y	Y	Y	U	Y	Y	Moderate	—
23	Kalata et al. [9] (2025)	Soccer/Athletics	CS	Y	Y	Y	Y	Y	Y	Y	Y	Low	—
24	Domínguez-Navarro et al. [31] (2024)	Basketball	CS	Y	Y	Y	Y	Y	Y	Y	Y	Low	—
25	Nikityuk [32] (2025)	Multi-sport	CS	Y	Y	U	Y	N	N	U	Y	High	*Stabilometric methodology with limited reliability data; confounder adjustment not reported.*

Domains (D1–D8) refer to the eight items of the JBI Critical Appraisal Checklist for Analytical Cross-Sectional Studies:
D1 = inclusion criteria clearly defined; D2 = study subjects and setting described in detail; D3 = exposure measured in a valid and reliable way; D4 = objective, standard criteria used for measurement of the condition; D5 = confounding factors identified; D6 = strategies to deal with confounding stated; D7 = outcomes measured in a valid and reliable way; D8 = appropriate statistical analysis used.

Design key: CS = analytical cross-sectional; PC = prospective cohort; LC = longitudinal cohort. For PC/LC studies the JBI 11-item cohort checklist was used; items 1–7 align with D1–D7 above and items 8–11 (follow-up reporting, completeness, handling of loss to follow-up) are summarized in the Notes column.

Cell key: Y = Yes (criterion met); U = Unclear; N = No (criterion not met). Overall RoB judgements (Low / Moderate / High) follow standard JBI conventions based on the number and severity of unmet items.

Table S4. GRADE Summary of Findings for the four quantitative synthesis domains

Table S4. GRADE evidence profile and Summary of Findings table for the four pre-specified quantitative synthesis domains. Because the body of evidence is predominantly observational, the starting certainty rating was Low and was further downgraded on a domain by domain basis (risk of bias, inconsistency, indirectness, imprecision, publication bias). The plain language interpretation column translates the pooled estimate and its certainty into a statement that is directly applicable to clinical and training-related decision-making.

Outcome	Studies / participants	Pooled estimate (95% CI; heterogeneity)	GRADE downgrading judgements	Certainty	Plain-language interpretation
Magnitude of single-leg countermovement jump (SLCMJ) asymmetry, expressed as % between-limb difference	9 studies; N = 2,344 youth athletes	Pooled mean asymmetry: 10.8% 95% CI 6.7 to 14.9 $\tau^2 = 19.29$; $I^2 = 78\%$; prediction interval not reported (k < 10)	Risk of bias: Not serious - most contributing studies at low/moderate RoB; one study (Dol et al.) at high RoB but its exclusion in leave-one-out did not materially alter the pooled estimate.<br>Inconsistency: Serious — $I^2 = 78\%$; heterogeneity primarily explained by sport-specific demands and	VERY LOW	*Inter limb SLCMJ asymmetry of ~10% appears to be a normative adaptation in youth athletes, with the magnitude meaningfully modified by sport specific demands; universal interpretive thresholds are not supported by the present evidence.*

Outcome	Studies / participants	Pooled estimate (95% CI; heterogeneity)	GRADE downgrading judgements	Certainty	Plain-language interpretation
			methodological diversity (force-plate vs field-based jump assessment).<br>Indirectness: Not serious — outcome directly aligned with population (youth athletes) and construct (unilateral vertical jump asymmetry).<br>Imprecision: Serious — the Hartung–Knapp 95% CI spans ≈8 percentage points and the per-study estimates range widely (≈5–21%), reflecting substantial between-study dispersion.<br>Publication bias: Undetected — Egger's intercept 0.32 (95% CI −0.84 to 1.48; p = 0.58); trim-and-fill imputed no studies (Figure S1).		
Association between SLCMJ asymmetry and linear sprint performance, expressed as Pearson r (Fisher's z-pooled)	4 studies; N = 200 youth athletes	Pooled r = −0.27<br>95% CI −0.55 to 0.07<br>I^2 = 48%; Q(3) = 5.79; p = 0.12	Risk of bias: Not serious — contributing studies at low/moderate RoB; reported correlations extracted directly with no imputation required.<br>Inconsistency: Serious — substantial between-study variability (volleyball r = −0.47 versus tennis r ≈ 0); driven by sport-specific neuromuscular demands.<br>Indirectness: Not serious — outcome (linear sprint performance) directly relevant to the asymmetry construct.<br>Imprecision: Serious — only 4 studies contribute and the pooled 95% CI crosses the null; the practical lower and upper bounds (small negative to negligible positive correlation) imply different clinical interpretations.<br>Publication bias: Could not be formally assessed (k < 10); qualitative inspection did not suggest selective reporting.	VERY LOW	*Static SLCMJ asymmetry shows at most a small, sport dependent association with linear sprint performance and is unlikely to be useful as a stand-alone predictor of sprint capacity in youth athletes.*
Magnitude of time-based change-of-direction (COD) asymmetry, expressed as % between-limb difference	4 studies; N = 681 youth athletes	Pooled mean asymmetry: 7.4%<br>95% CI 0.5 to 14.2<br>I^2 = 64%; Q(3) = 8.33; p = 0.04	Risk of bias: Not serious — contributing studies at low/moderate RoB; harmonized time-based metric used consistently.<br>Inconsistency: Serious — moderate-to-substantial heterogeneity (I^2 = 64%) explained by unilateral- vs bilateral-demand sport classification (handball ≈13% vs tennis ≈4% and mixed ≈5%).<br>Indirectness: Not serious — outcome directly relevant to sport-specific functional performance.<br>Imprecision: Serious — only 4 studies contribute; pooled estimate stable but precision limited by small study count.<br>Publication bias: Could not be formally assessed (k < 10); Begg's test	VERY LOW	*Time-based COD asymmetry of approximately 6% appears typical for youth team sport athletes, with handball populations consistently higher; interpretation should remain sport-conditional and metric specific (deficit-based and iso-inertial COD measures are not interchangeable).*

Outcome	Studies / participants	Pooled estimate (95% CI; heterogeneity)	GRADE downgrading judgements	Certainty	Plain-language interpretation
			p = 0.31; visual inspection showed sport-specific clustering rather than asymmetry from selective reporting.		
Standardized effect of biological maturation stage on asymmetry magnitude (pooled across pre-/circa-/post-PHV contrasts)	5 studies; N = 1,509 youth athletes	Pooled standardized effect: Cohen's d = 0.35 95% CI 0.18 to 0.52; I² = 42%	Risk of bias: Not serious — contributing studies include one large prospective cohort and one 6-year longitudinal cohort, both judged at low RoB. Starting certainty for this outcome was set at moderate rather than low, reflecting the prospective longitudinal cohort designs of the contributing studies; the single imprecision downgrade therefore yields a final rating of low.<br>Inconsistency: Not serious — heterogeneity moderate (I² = 42%) and consistent with substantive between-sport variability rather than methodological artefact.<br>Indirectness: Not serious — outcome aligns with the developmental question; PHV operationalization broadly comparable across studies.<br>Imprecision: Serious — only 5 contributing studies; the magnitude (small-to-moderate effect) is precisely estimated but the body of evidence is small.<br>Publication bias: Could not be formally assessed (k < 10); no qualitative signal of selective reporting identified.	LOW	*Biological maturation status (operationalized via PHV) is associated with a small-to-moderate increase in asymmetry magnitude, with the highest values observed during the circa-PHV phase; maturation based rather than age based monitoring is supported by the evidence.*

Starting certainty rating: low (observational evidence). One level downgrades applied where a domain was judged serious; two level downgrades reserved for very serious concerns. For outcomes informed predominantly by prospective longitudinal cohort designs (the maturation stage contrast), the starting certainty was set at moderate rather than low, in line with GRADE guidance for higher quality observational designs.

Table S5. Full search syntax for each electronic database

Table S5. Database-specific search syntax, last searched dates, and filters/limits used to retrieve records for screening. All searches combined three keyword blocks: (1) asymmetry/laterality; (2) youth population; (3) sport/performance context. Searches were limited to peer-reviewed journal articles published in English, with no lower date limit (database inception) and an upper limit of 31 October 2025. Reference lists of included studies and relevant reviews were screened manually to identify additional records.

Database	Date last searched	Filters and limits applied	Search syntax
MEDLINE (via PubMed)	23 October 2025	Filters: Humans; English; Journal Article; publication date inception–31/10/2025. Mesh terms used where available; Title/Abstract field tags applied.	#1 ("inter-limb asymmetry"[tiab] OR "interlimb asymmetry"[tiab] OR "limb symmetry index"[tiab] OR "bilateral imbalance"[tiab] OR "between limb difference"[tiab] OR "side to side difference"[tiab] OR laterality[tiab] OR "symmetry angle"[tiab] OR "dominant limb"[tiab])<br>#2 ("Child"[Mesh] OR "Adolescent"[Mesh] OR child*[tiab] OR adolescen*[tiab] OR youth[tiab] OR pediatric*[tiab] OR

Database	Date last searched	Filters and limits applied	Search syntax
			paediatric*[tiab] OR junior*[tiab] OR "young athlete*"[tiab] OR pubescent[tiab] OR prepubescent[tiab])<br>#3 ("Sports"[Mesh] OR "Athletic Performance"[Mesh] OR sport*[tiab] OR athlet*[tiab] OR sprint*[tiab] OR jump*[tiab] OR "change of direction"[tiab] OR plyometric*[tiab] OR maturation[tiab] OR "peak height velocity"[tiab])<br>#4 #1 AND #2 AND #3<br>Limits applied: Humans; English; Journal Article
Scopus	23 October 2025	Fields: TITLE-ABS-KEY. Document type: ar (article). Source type: journal. Language: English.	(TITLE-ABS-KEY ("inter-limb asymmetry" OR "interlimb asymmetry" OR "limb symmetry index" OR "bilateral imbalance" OR "between-limb difference" OR "side-to-side difference" OR laterality OR "symmetry angle" OR "dominant limb"))<br>AND<br>(TITLE-ABS-KEY (child* OR adolescen* OR youth OR pediatric* OR paediatric* OR junior OR "young athlete*" OR pubescent OR prepubescent))<br>AND<br>(TITLE-ABS-KEY (sport* OR athlet* OR sprint* OR jump* OR "change of direction" OR plyometric* OR maturation OR "peak height velocity" OR PHV))<br>AND (DOCTYPE (ar))<br>AND (SRCTYPE (j))<br>AND (LANGUAGE (english))
Web of Science Core Collection	23 October 2025	Indexes: SCI-EXPANDED, SSCI, ESCI. Field tag: TS (Topic = Title, Abstract, Author Keywords, Keywords Plus). Document Type: Article. Languages: English.	#1 TS=("inter-limb asymmetry" OR "interlimb asymmetry" OR "limb symmetry index" OR "bilateral imbalance" OR "between-limb difference" OR "side-to-side difference" OR laterality OR "symmetry angle" OR "dominant limb")<br>#2 TS=(child* OR adolescen* OR youth OR pediatric* OR paediatric* OR junior OR "young athlete*" OR pubescent OR prepubescent)<br>#3 TS=(sport* OR athlet* OR sprint* OR jump* OR "change of direction" OR plyometric* OR maturation OR "peak height velocity" OR PHV)<br>#4 #1 AND #2 AND #3<br>Refined by: DOCUMENT TYPES = Article; LANGUAGES = English
SPORTDiscus (via EBSCOhost)	23 October 2025	Field tag: AB OR TI OR SU. Document type: Academic Journal. Language: English.	S1 AB ("inter-limb asymmetry" OR "interlimb asymmetry" OR "limb symmetry index" OR "bilateral imbalance" OR "between-limb difference" OR "side-to-side difference" OR laterality OR "symmetry angle")<br>S2 AB (child* OR adolescen* OR youth OR pediatric* OR paediatric* OR junior OR "young athlete*" OR pubescent OR prepubescent)<br>S3 AB (sport* OR athlet* OR sprint* OR jump* OR "change of direction" OR plyometric* OR maturation OR "peak height velocity")<br>S4 S1 AND S2 AND S3<br>Limiters: Academic Journals; English Language
Semantic Scholar (API + interface)	23 October 2025	Fields: Title and Abstract. Filters: peer-reviewed; English. Multi-query merge across the three keyword blocks; deduplication by DOI.	Combined query (across title and abstract):<br>("inter-limb asymmetry" | "interlimb asymmetry" | "limb symmetry index" | "bilateral imbalance" | "between-limb difference" | "side-to-side difference" | laterality)

Database	Date last searched	Filters and limits applied	Search syntax
			AND (child OR adolescent OR youth OR junior OR pediatric OR paediatric OR pubescent OR prepubescent OR "young athletes") AND (sport OR athlete OR sprint OR jump OR "change of direction" OR plyometric OR maturation OR "peak height velocity") Filters applied: language = English; peer-reviewed sources.
OpenAlex (API + interface)	23 October 2025	Search across title and abstract. Filters: type = article; language = English; from-publication-date = 1900-01-01, to-publication-date = 2025-10-31.	Search: ("inter-limb asymmetry" OR "interlimb asymmetry" OR "limb symmetry index" OR "bilateral imbalance" OR "between-limb difference" OR "side-to-side difference" OR laterality) AND (child OR adolescent OR youth OR junior OR pediatric OR paediatric OR pubescent OR prepubescent OR "young athletes") AND (sport OR athlete OR sprint OR jump OR "change of direction" OR plyometric OR maturation OR "peak height velocity" OR PHV) Filters: type: journal-article AND language:en AND from-publication-date:1900-01-01 AND to-publication-date:2025-10-31

Figure S1. Funnel plot for the SLCMJ asymmetry meta-analysis

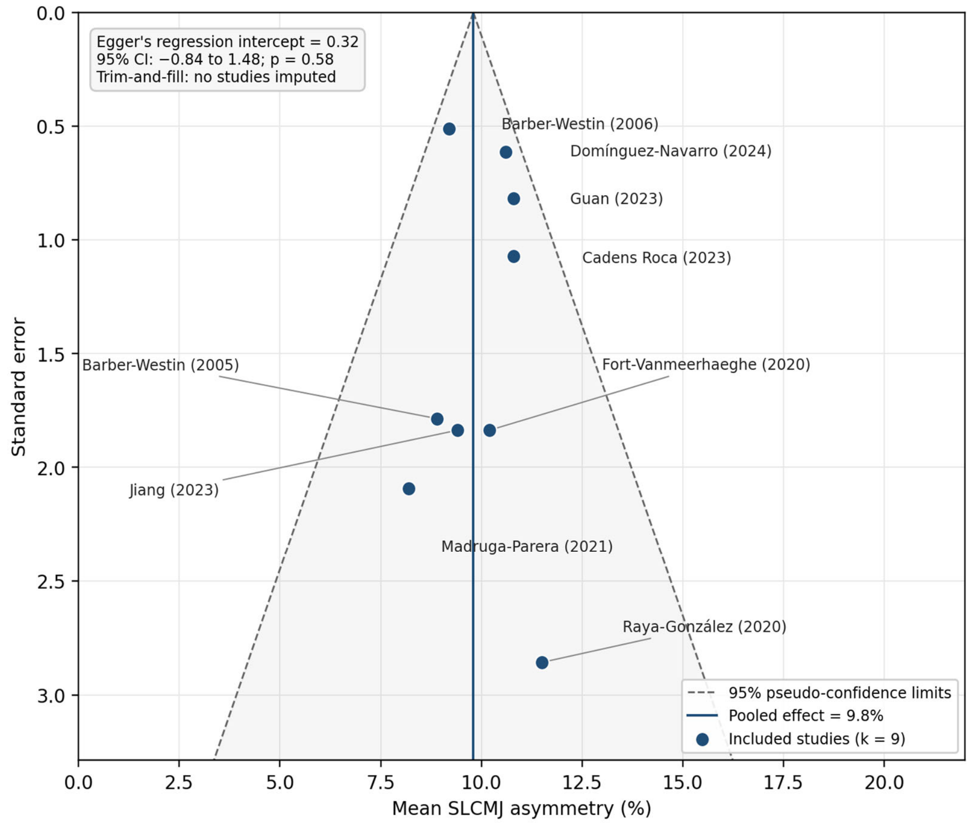

Figure S1. Funnel plot for the nine studies contributing to the pooled estimate of single-leg countermovement jump (SLCMJ) asymmetry. The vertical axis displays the standard error of each study's mean asymmetry estimate (inverted, as is conventional for funnel plots) and the horizontal axis displays the mean SLCMJ asymmetry expressed as a percentage. The solid vertical line denotes the random-effects pooled estimate (10.8%); the dashed

lines indicate the 95% pseudo-confidence envelope around the pooled estimate. Egger's regression intercept was 0.32 (95% CI −0.84 to 1.48; $p = 0.58$), and trim and fill imputed no missing studies, providing no statistical evidence of small study or publication bias for this synthesis. The plot should be interpreted alongside the qualitative GRADE judgements presented in Table S4.
